# Purification and Characterization of the Pink-Floyd Drillipeptide, a Bioactive Venom Peptide from *Clavus davidgilmouri* (Gastropoda: Conoidea: Drilliidae)

**DOI:** 10.3390/toxins12080508

**Published:** 2020-08-07

**Authors:** Victor M. Chua, Joanna Gajewiak, Maren Watkins, Samuel S. Espino, Iris Bea L. Ramiro, Carla A. Omaga, Julita S. Imperial, Louie Paolo D. Carpio, Alexander Fedosov, Helena Safavi-Hemami, Lilibeth A. Salvador-Reyes, Baldomero M. Olivera, Gisela P. Concepcion

**Affiliations:** 1 The Marine Science Institute, University of the Philippines, Diliman, Quezon City 1101, Philippines; vmchua28@gmail.com (V.M.C.); ilramiro@up.edu.ph (I.B.L.R.); carlaomaga@yahoo.com (C.A.O.); luipaolo@gmail.com (L.P.D.C.); lsreyes@msi.upd.edu.ph (L.A.S.-R.); 2 School of Biological Sciences, University of Utah, Salt Lake City, UT 84112, USA; jgajewiak@gmail.com (J.G.); maren.watkins@hsc.utah.edu (M.W.); samespino@gmail.com (S.S.E.); imperial@biology.utah.edu (J.S.I.); safavihelena@sund.ku.dk (H.S.-H.); olivera@biology.utah.edu (B.M.O.); 3 Department of Biomedical Sciences, University of Copenhagen, 2200 Copenhagen N, Denmark; 4 Severstov Institute of Ecology and Evolution, Russian Academy of Sciences, Leninsky prospect 33, Moscow 119071, Russia; fedosovalexander@gmail.com; 5 Department of Biochemistry, University of Utah, Salt Lake City, UT 84112, USA

**Keywords:** venom, *Clavus*, Drilliidae, drillipeptide

## Abstract

The cone snails (family Conidae) are the best known and most intensively studied venomous marine gastropods. However, of the total biodiversity of venomous marine mollusks (superfamily Conoidea, >20,000 species), cone snails comprise a minor fraction. The venoms of the family Drilliidae, a highly diversified family in Conoidea, have not previously been investigated. In this report, we provide the first biochemical characterization of a component in a Drilliidae venom and define a gene superfamily of venom peptides. A bioactive peptide, cdg14a, was purified from the venom of *Clavus davidgilmouri* Fedosov and Puillandre, 2020. The peptide is small (23 amino acids), disulfide-rich (4 cysteine residues) and belongs to the J-like drillipeptide gene superfamily. Other members of this superfamily share a conserved signal sequence and the same arrangement of cysteine residues in their predicted mature peptide sequences. The cdg14a peptide was chemically synthesized in its bioactive form. It elicited scratching and hyperactivity, followed by a paw-thumping phenotype in mice. Using the Constellation Pharmacology platform, the cdg14a drillipeptide was shown to cause increased excitability in a majority of non-peptidergic nociceptors, but did not affect other subclasses of dorsal root ganglion (DRG) neurons. This suggests that the cdg14a drillipeptide may be blocking a specific molecular isoform of potassium channels. The potency and selectivity of this biochemically characterized drillipeptide suggest that the venoms of the Drilliidae are a rich source of novel and selective ligands for ion channels and other important signaling molecules in the nervous system.

## 1. Introduction

The successful evolutionary radiation of the venomous marine snails in the superfamily Conoidea has generated a significant fraction of the biodiversity of living mollusks (>20,000 species) [1,2]. Traditionally, three groups of gastropods, the cone snails, the auger snails, and the turrids, were thought to comprise this superfamily. However, anatomical and molecular phylogenetic studies revealed that while cone snails belong to a single monophyletic lineage (the family Conidae), and the auger snails can similarly be grouped into a single clade (the family Terebridae), the conoidean snails that have generally been referred to as turrids (the former family Turridae, *sensu lato*) comprise several phylogenetically unrelated groups, some of which are more closely related to the cone snails than to the type genus of the Turridae, *Turris* [3,4,5].

In a proposed reorganization of the superfamily Conoidea based on molecular data with the most recent emendations [6], the members of the old family Turridae have been reclassified into 15 monophyletic families. One of the most diverse phylogenetic lineages of former “turrids” whose distinctiveness has long since been acknowledged, is the family Drilliidae [1,6]. The venoms of this species-rich conoidean family until recently remained completely uncharacterized, likely because most drilliids are relatively small, uncommon, and predominantly collected offshore. Earlier this year, results of the transcriptomic analysis of two species in the genus *Clavus* were published [7] featuring unparalleled diversity of putative venom components in these gastropods. An important finding of Lu et al. [7] was the high number of transcripts encoding short cysteine-rich mature peptides, sharing some crucial structural features of conotoxins [8]. Such structural similarity suggests that drilliid venom peptides are also likely to share some pharmacological properties of conotoxins. Taking into account the broad interest in conotoxins, discovering a principally novel source of natural peptides with similar bioactivity would be of great importance. However, first, the predicted transcripts need to be validated by the analysis of venom proteins, and second, the proposed bioactivity has to be demonstrated.

**Figure 1:**
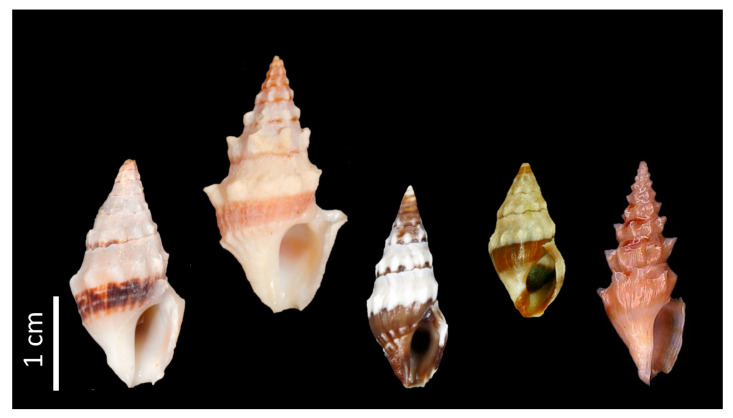
Shells of Clavus species. From left to right: Clavus davidgilmouri, Clavus canalicularis, Clavus viduus, Clavus unizonalis, and Clavus suduirauti [9,10,11].

In this report, we describe the purification and characterization of cdg14a, isolated from the venom of *Clavus davidgilmouri* Fedosov and Puillandre, 2020, the first bioactive peptide of any species in the family Drilliidae. Following Lu et al. [7], we refer to the venom peptides from species in this family as “drillipeptides” to distinguish these from conopeptides and turripeptides. The species that we have investigated, *Clavus davidgilmouri*, measures ~15–30 mm (Figure 1); it is one of the species in the broadly distributed Indo-Pacific *Clavus canalicularis* complex, whose taxonomy has been recently revised [9] and for which transcriptomic data is available [7].

## 2. Results

### 2.1. Identification and Initial Characterization of Drillipeptide cdg14a

**Figure 2:**
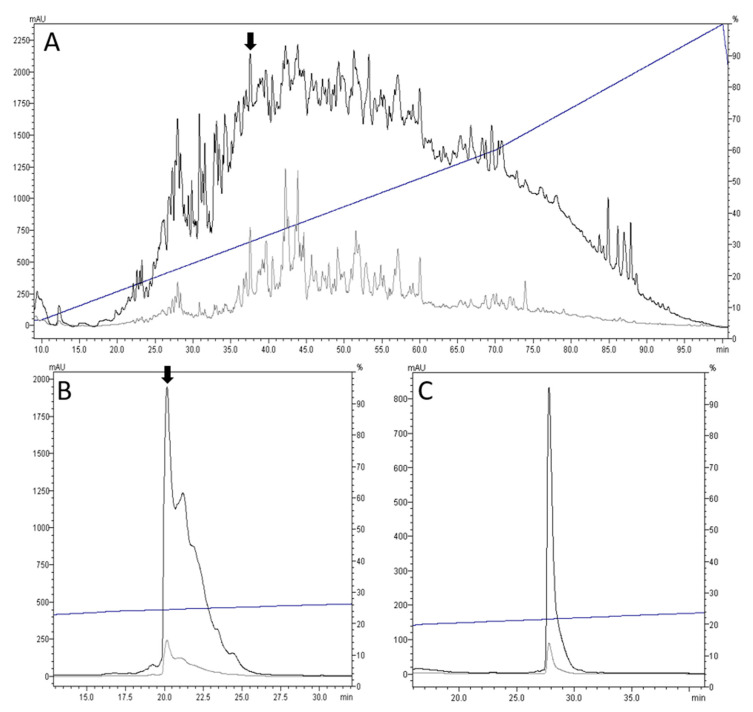
Purification of cdg14a by high-performance liquid chromatography (HPLC) using C18 semi-preparative and analytical columns. The elution profiles show two different wavelengths used: 220 nm (black) and 280 nm (grey), with the elution gradient indicated by a blue line. (**A**) Chromatogram of the crude venom extract using a C18 semi-preparative column with linear gradient ranging from 6% to 60% solvent B (90% acetonitrile with 0.1% trifluoroacetic acid) at 0.9% solvent B/min, followed by 60% to 100% at 1.3% solvent B/min. The peak of the bioactive fraction is indicated by an arrow. (**B**) Chromatogram of the bioactive fraction reinjected in the HPLC using a C18 analytical column run at a shallower linear gradient ranging from 23% to 27% solvent B at 0.15% solvent B/min. The peak of the subfraction containing the bioactive peptide cdg14a is indicated by an arrow. (**C**) Chromatogram of the purified peptide cdg14a, showing a single, symmetrical peak.

The chromatographic profile of the crude venom is shown in Figure 2A; bioactivity was detected in the fraction indicated by the arrow in Figure 2A,B. This fraction caused a shaking phenotype in mice, followed by scratching, excessive grooming, and hind feet tapping. This bioactivity was used to guide the purification of the active component, and a high-performance liquid chromatography (HPLC) profile of the purified venom drillipeptide is shown in Figure 2C.

**Figure 3:**
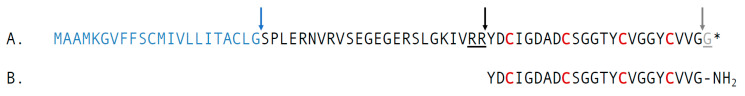
(**A**) CvDg14.4, cdg14a precursor sequence. Blue arrow indicates boundary between signal sequence and propeptide, black arrow between propeptide and mature toxin region, gray arrow between mature toxin and post-peptide region. Underlined residues denote recognition sites for predicted propeptide and post-peptide cleavage sites. The stop codon, denoted by an asterisk (*), is post-translationally modified to NH_2_, as shown in Figure 3B. (**B**) Peptide sequence of cdg14a.

The sequence of the analyzed peptide was deduced from the precursor sequence identified in the transcriptome (Figure 3A); the apparently homogenous peptide was analyzed by mass spectrometry, and its peptide sequence (Figure 3B) matched the one predicted from transcriptome. The 23 AA peptide has 4 Cys residues, in an arrangement similar to Framework XIV of conotoxins (—C—C—C—C—), first characterized from peptides of the J-conopeptide superfamily [12]. We refer to this peptide as cdg14a, belonging to the J-like drillipeptide superfamily (a nomenclature consistent with that proposed for venom peptides from conoideans) [13].

### 2.2. Characterization of the Precursor of cdg14a

**Figure 4:**
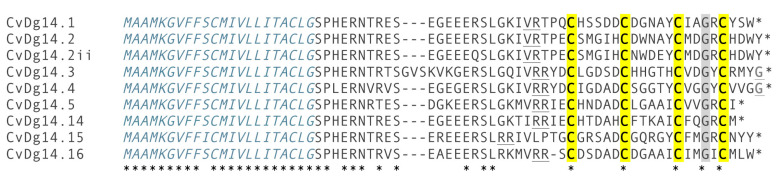
*Clavus davidgilmouri* complete precursor sequences related to cdg14A aligned with MUSCLE (MacVector 17.0) and refined by eye. These sequences belong to a single family of genes as indicated by their highly conserved N-terminal signal sequence (indicated in italics). Cysteines are highlighted in yellow and the conserved glycine in grey. Predicted precursor and post-peptide cleavage sites are underlined. Conserved amino acid residues are denoted by an asterisk (*) below the alignment.

The encoded translation product has the organizational characteristics of conopeptide precursors (Figure 3A) comprising a signal sequence at the N-terminus, followed by a propeptide region, and the mature peptide region at the C-terminal end. The arrows indicate the boundaries between signal sequence and propeptide, as well as between the propeptide and the mature peptide region. A transcriptome analysis of two individual specimens [7] revealed at least nine related peptide precursors that share a highly conserved signal sequence (Figure 4), defining a gene superfamily, with all members found in *Clavus davidgilmouri* also sharing the same arrangement of Cys residues (Framework XIV) in the mature toxin region; the analysis also revealed the presence of a conserved glycine (Gly) in the third inter-cysteine loop.

### 2.3. Chemical Synthesis of cdg14a

**Figure 5:**
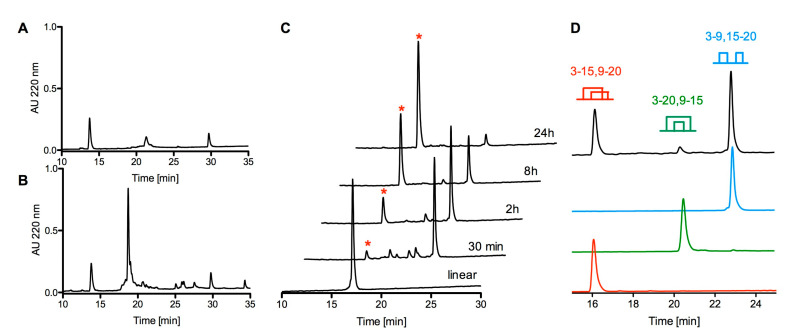
Chemical synthesis and folding of cdg14a. (**A**) HPLC chromatogram of a crude linear cdg14a eluted on an analytical C18 column using a gradient ranging from 5% to 65% solvent B in 30 min with 1 mL/min flow rate. (**B**) HPLC chromatogram of a crude linear cdg14a modified with 2-aminoethyl methanethiosulfonate hydrobromide (MTSEA) (HPLC conditions are the same as in panel A). (**C**) oxidative folding of cdg14a monitored by HPLC, using a C18 column and a gradient ranging from 15% to 45% solvent B, with a flow rate 1 mL/min. Single asterisk denotes the native-like fold of cdg14a. (**D**) zoom-in on 4 h oxidative folding time point (black HPLC trace) with all possible folding isomers identified at 220 nm. The red trace represents the native folding isomer with the globular-like connectivity Cys3-Cys15, Cys9-Cys20; green trace represents ribbon-like folding isomer with the Cys3-Cys20, Cys9-Cys15 connectivity; blue trace represents the fast folding product with the bead-like connectivity Cys3-Cys9, Cys15-Cys20. The traces were collected using the same gradient as described for panel (**C**).

In order to verify that the biochemically characterized peptide is indeed responsible for the activity detected in the venom fraction, cdg14a was chemically synthesized as described under the Methods section. The linear form of cdg14a was problematic to handle due to its low solubility; therefore, to increase its hydrophilicity, the cysteines of the peptide were temporarily modified using 2-aminoethyl methanethiosulfonate hydrobromide (MTSEA), a positively charged sulfhydryl-selective reagent that forms mixed disulfides with the cysteine residues [14]. Figure 5 illustrates profiles of the crude peptide solution (the same volume and concentration) injected on the C18 column before (Figure 5A) and after the MTSEA treatment (Figure 5B). This approach allowed us to obtain 3378 nmols of the linear, SEA-modified peptide (SEA, 2-aminoethanethiol) out of 108 mg of the cleaved resin, with purity ranging from 64% to 81%. Electrospray Ionization (ESI) mass spectrometry analysis confirmed the identity of cdg14a-SEA: calculated (M) = 2574.92 Da; determined (M) = 2574.92 Da. This method was recently successfully employed to obtain conopeptide AtVIA (V28L:T30S) [15].

The linear cdg14a-SEA was then subjected to an oxidative folding reaction in the presence of a 1:1 mixture of reduced and oxidized glutathione in a buffered solution (pH = 7.5). Two-step transformation was observed: a rapid folding step leading to a more hydrophobic isomer than the native peptide (maximum accumulation of the product in the first 30 min of the reaction) and a slow reshuffling step (in over 24 h) into the final product, with the same retention time of the native peptide (Figure 5C). The significant difference in the retention times between both folding forms (6 min) suggests that the peptide initially collapsed to an isomer with the hydrophobic residues exposed on its surface, which then were moved toward its core during the long reshuffling process. Following the 24-h folding protocol, cdg14a was obtained with 98% purity in 44% yield (based on the linear peptide used), whereas the 30-min protocol led to the more hydrophobic isomer, cgd14a_pk2, in 26% yield and 95% purity. MALDI mass spectrometry confirmed the identity of both peptides. For cdg14a, the major mass determined, 2293.92 Da, was a sodium adduct, whereas the expected mass (M + H)^+^ = 2271.91 Da was a minor signal (calculated (M + H)^+^ = 2271.84 Da). For cdg14a_pk2, an additional potassium adduct was observed with a mass of 2309.87 Da.

**Figure 6:**
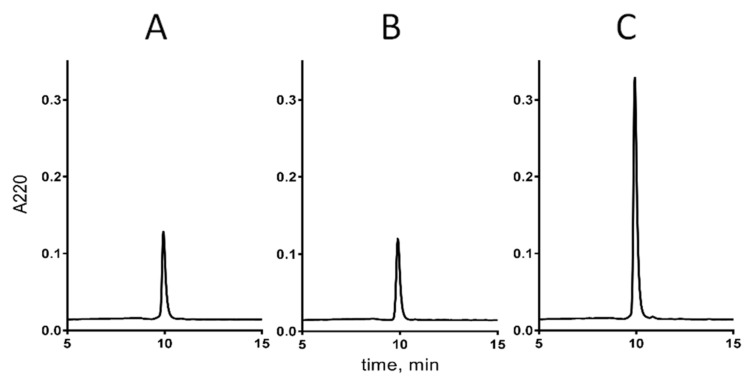
Coelution profile of native and synthetic cdg14a. The HPLC runs were carried out using an analytical C18 column and a gradient of 0.15% solvent B/min. (**A**) Native cdg14a isolated from *Clavus davidgilmouri* venom. (**B**) Synthetic cdg14a. (**C**) Mixture of the native and synthetic samples (1:2) of cdg14a.

The synthetic cdg14a peptide was obtained in the glutathione-assisted folding, and the native peptide was subjected to the co-elution experiment, the results of which are illustrated in Figure 6. Both peptides were first eluted individually (Figure 6A,B) and then as a mixture in 1:2 ratio (Figure 6C) of the native to the synthetic peptide. The individual peaks had the same retention times, and the mixed peak showed a uniform profile. 

To establish connectivity of the native peptide, an orthogonal protection of the cysteines and a stepwise oxidative folding were employed. The globular (Cys3–15, Cys9–20), bead (Cys3–9, Cys15–20), and ribbon (Cys3–20, Cys9–15) forms of cdg14a were synthesized following a two-step oxidative folding: first in the presence of reduced and oxidized glutathione to form the first disulfide bridge, followed by folding in the presence of iodine to facilitate the second disulfide bond formation. Based on the co-elution experiments with the peptide obtained in a one-step, glutathione-assisted folding experiment (which co-eluted with the native peptide) and the product of a two-step directed folding (Figure S5, Supplementary Information), it was established that the native peptide has the globular (Cys3–15, Cys9–20) fold, and the kinetically favored form, cdg14a_pk2, has the bead (Cys3–9, Cys15–20) fold (Figure 5D). Both the bead and the ribbon forms were prone to precipitation from the solution, and re-solubilization was not achieved, therefore inhibiting assessment of their bioactivity.

### 2.4. Behavioral Assay of cdg14a

The native peptide (~2 nmol) caused shaking in mice, which was followed by scratching, excessive grooming, and foot tapping. When injected intracranially into mice, the synthetic cdg14a elicited the same bioactivity as the native peptide at 2.5 nmol and higher. Because of the extremely limited amounts of the native peptide, the synthetic peptide was used to characterize further the in vivo biological activity.

The synthetic peptide elicited a series of distinctive behavioral phenotypes in mice in a sequential combination not previously observed for any conopeptide; particularly noteworthy was the thumping phenotype. At a dose of 2.5 nmol, the peptide elicited a shaking phenotype, scratching with the hind paws, and excessive grooming; this was followed by a characteristic thumping of the hind paws. The onset and duration of each symptom were dependent on the dose (Table 1).

### 2.5. Constellation Pharmacology

Cdg14a induces aberrant behavior in mice upon intracranial injection suggesting that this peptide is active in the murine central nervous system (CNS). We next determined whether the peptide was also active in the peripheral nervous system (PNS). To achieve this goal, we performed calcium imaging experiments on dissociated dorsal root ganglion (DRG) neurons harvested from mice. The neuronal subclass affected by the peptide was determined by the response of neurons to the following pharmacological agents: allyl isothiocyanate (TrpA1 agonist), menthol (TrpM8 agonist) and capsaicin (TrpV1 agonist). 

**Figure 7:**
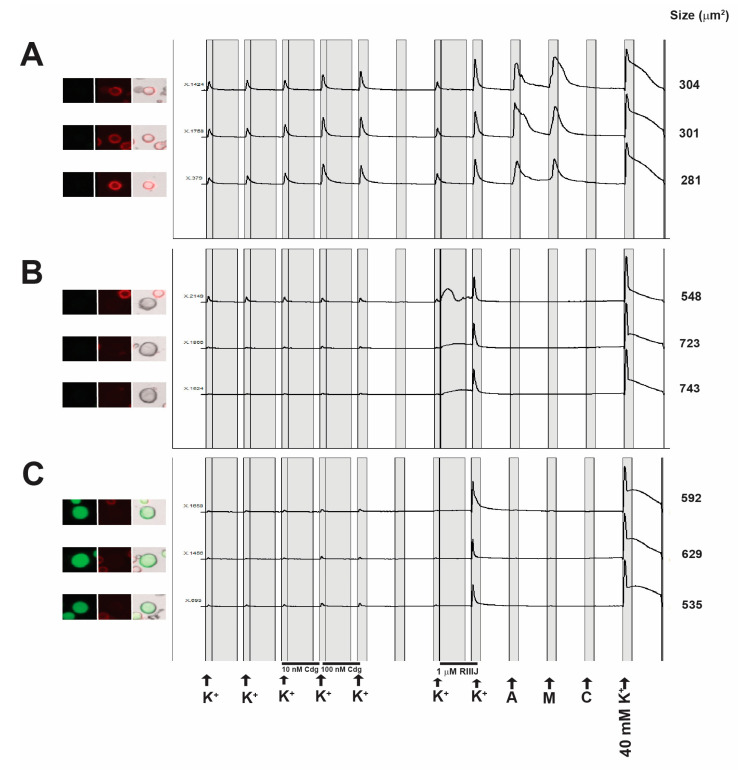
Constellation Pharmacology. The images starting from the left are taken using (1) the green fluorescent (GFP) filter to visualize calcitonin gene related peptide (CGRP), (2) Cy5 filter to visualize cells stained with isolectin B4 (IB4), (3) merged images of the GFP, Cy5 and brightfield channels, and (4) the calcium imaging trace. Each calcium imaging trace corresponds to the response of a single cell to the pharmacological challenge indicated on the *X*-axis at the bottom of panel C. The *Y*-axis indicates the ratio of emission at 340/380 nm. The sizes of each cell are indicated on the right of each trace. The pharmacological agents are as follows: allyl isothiocyanate (**A**), menthol (M) and capsaicin (**C**). Panel (**A**) is cell class affected by cdg14a. These cells are small (<500 μm^2^), with strong IB4 staining, and respond to allyl isothiocyanate, an agonist of the TrpA1 receptor and to menthol, an agonist of TrpM8. The effects of cdg14a at different concentrations are seen as higher peak amplitude in pulses 4 to 5 relative to baseline responses in pulses 1 to 3. The mean response to 10 nM cdg is 76 ± 11%, increase in peak height relative to the control. The K^+^ concentration in these pulses is 20 mM unless specified. Panels (**B**,**C**) are classes of cells that are generally not affected by cdg14a. Cells in (**B**) are large cells (>500 μm^2^) that show a direct response to the voltage-gated K^+^ channel inhibitor conotoxin κM-RIIIJ (RIIIJ), while cells in (**C**) (>500 μm^2^) which are CGRP positive, have an indirect response to RIIIJ as shown by a higher response to 20 mM K^+^ immediately after RIIIJ incubation.

The application of synthetic cdg14a amplified the response of the cells to depolarization induced by the application of 20 mM KCl (Figure 7). Interestingly, a significant effect of cdg14a is seen in a class of small diameter neurons (<500 μm^2^) that are labeled by isolectin B4 (IB4), a plant lectin that binds the carbohydrate moiety of glycoproteins and glycolipids. Further, this cell class responds to the transient receptor Ankyrin 1 (TrpA1) agonist allyl isothiocyanate (AITC) and menthol, an agonist of the transient receptor potential cation channel subfamily M (melastin) member 8 (TrpM8). Experiments on DRG neurons harvested from four mice showed that 10 nM cdg14a induces a 76 ± 11% maximal response to 20 mM K^+^ in the presence of the peptide relative to control in this neuronal class.

The activity observed in DRG neurons is similar to that of the voltage-gated potassium channel (VGKC) blocker peptide cce9b from another conoidean, *Crassispira cerithina* [16], suggesting that, like cce9b, cdg14a possibly inhibits a subset of VGKC by either direct or indirect inhibition of channel activity. The spectrum of DRG neurons affected is unique and not observed with any other ligand tested so far.

## 3. Discussion

This is the first report describing the characterization of any drilliid venom component. We identified and characterized a bioactive peptide from the venom of *Clavus davidgilmouri*, cdg14a, and defined a putative new gene superfamily, the J-like drillipeptides, comprising a diversity of venom peptides structurally similar to cdg14a and only known from the species of Drilliidae. We confirmed previous results of Lu et al. [7] that several different J-like drillipeptides are expressed in the venom gland of *Clavus davidgilmouri.*

Like conopeptides, the drillipeptides are relatively small and rich in disulfides (4 Cys residues out of 23AA in cdg14a). The organization of the J-like drillipeptide precursors is directly comparable to the conopeptides—three well-defined regions can readily be identified: a signal sequence that is highly conserved, a propeptide region, and a mature peptide region at the C-terminus (Figure 4). Although they share the same Cys framework (Framework XIV, —C—C—C—C—), there appears to be little sequence similarity between the J-conopeptide superfamily and the J-like drillipeptide superfamily. The J-conopeptide superfamily precursor sequences obtained from the Conoserver database and those of the J-like drillipeptide superfamily show 12.8–23.1% identity, whereas the pairwise identities are 53.8–79.5% among the J-conopeptide precursors and 76.7–93.3% among J-like drillipeptide precursors. The J-like drillipeptides also show predicted C-terminal amidation in the mature peptide, a modification that is fairly common in conopeptides, including the J superfamily.

The drillipeptide cdg14a was chemically synthesized, and the linear peptide was folded into the native, biologically active conformation by oxidizing Cys residues to form disulfide bonds. The oxidation of the linear cdg14a peptide is rather unprecedented, in that a bead intermediate (with the cysteine connectivity 3–9, 15–20 different from the final one) is rapidly accumulated, followed by a slow conversion to an oxidized peptide that has the native, globular disulfide framework (with the cysteine connectivity 3–15, 9–20). This kinetically favored form elutes much later on a reverse-phase HPLC column, suggesting that hydrophobic residues are exposed to the medium to a much greater extent than in the native, biologically active peptide. The equilibration to the presumably lower energy native disulfide-bonded peptide is rather slow, as shown in Figure 5, under the standard folding conditions for conoidean venom peptides. Interestingly, the ribbon form of the peptide was also identified in the folding solution, possibly being the second folding intermediate before reshuffling to the native form (Figure 5D).

The isolated and characterized peptide, cdg14a, is novel in its biological activity. While the shaking and hyperactivity phenotype have been reported in several conopeptides that target potassium channels [17], calcium channels [18], and the neuronal noradrenaline receptor [19], the paw-thumping phenotype elicited after intracranial injection has never been observed for any venom peptide. Particularly striking as well is the sequence of behavioral phenotypes, which manifested at a dose-dependent time period after injection of the peptide: the mice became hyperactive and then exhibited the paw-thumping behavior. Since cdg14a was isolated from the venom of *Clavus davidgilmouri,* named after Mr. David Gilmour, CBE, a member of the band Pink Floyd, and the thumping phenotype was reminiscent of the Pink Floyd video featuring a long line of people thumping their feet, we refer to this peptide as the “Pink Floyd” peptide.

The peptide has also been characterized using calcium imaging combined with a pharmacological separation of different neuronal subtypes within the lumbar dorsal root ganglion of mice, a platform called Constellation Pharmacology [20]. The results of the constellation pharmacology analysis revealed that the peptide is surprisingly potent in mammalian systems, with nanomolar affinity for its targets. Furthermore, the cellular phenotypes observed are consistent with the highly selective inhibition of a specific subset of potassium channels present in about 20% of all of the dorsal root ganglion neurons. The non-peptidergic nociceptor neuronal population that does not express the CGRP peptide, and is labeled by isolectin IB4, is one of the major classes of neurons affected. After the peptide is applied, a greater increase in cytosolic (Ca)_i_ is observed after a standard pulse of KCl—this is consistent with the inhibition of a K^+^ channel present on the plasma membrane of these cells (although alternative explanations, such as increased activity of Na^+^ channels, are also possible).

Thus, the first drillipeptide characterized appears to have a unique profile of bioactivity, both at the whole animal behavioral level and at the cellular/molecular level. We are carrying out a more thorough characterization, including a comprehensive structure/function analysis. Since the J-like drillipeptide superfamily appears to be a rather large and diverse set of venom peptides within the genus *Clavus* [7], these may contribute significantly to the characterization of native ion channels present in different neurons. It has recently been established that different subclasses of neurons are likely to express different complements of ion channel isoforms [20,21,22,23]. Understanding the function and roles of these channels requires the availability of highly selective ligands for different ion channel isoforms, making the discovery of novel natural ligands with high selectivity to channel isoforms a high priority task. In this context, our finding that the J-like drillipeptide superfamily may provide numerous novel ion channel-targeted peptides is of great importance.

## 4. Materials and Methods 

### 4.1. Sample Collection

The specimens reported in this study and in the study of Fedosov and Puillandre, 2020 [9] came from the same sample collection. Species identification was confirmed by Alexander Fedosov. Forty-two live specimens of *Clavus davidgilmouri* were collected using tangle nets off Olango Island, Cebu, the Philippines. The snails were dissected on ice, and venom ducts were collected. For the transcriptome library assembly, 2 venom ducts were immediately suspended in RNAlater (Thermo Fisher Scientific, Waltham, MA, USA) and stored at −20 °C until RNA extraction. The remaining 40 venom ducts were pooled in micro-centrifuge tubes and frozen in liquid nitrogen. Upon arrival in the laboratory, venom ducts were transferred to and stored at −70 °C until venom peptide extraction. Specimens were collected under the gratuitous permit no. 0111–16 issued by the Department of Agriculture—Bureau of Fisheries and Aquatic Resources (DA-BFAR).

### 4.2. Venom Extraction and Purification

Venom ducts from *Clavus davidgilmouri* were thawed and re-suspended in 20% (*v*/*v*) acetonitrile (CH_3_CN) with 0.2% (*v*/*v*) trifluoroacetic acid (TFA) and cut into smaller pieces. The mixture was homogenized at 3000 RPM using a drill press and centrifuged at 15,000 RPM for 30 min. The supernatant liquid was collected and kept at −70 °C, while a second extraction of the pellet was done using 40% CH_3_CN with 0.2% TFA. The supernatants were pooled, diluted with 0.1% TFA, and applied to a semi-preparative C_18_ high-performance liquid chromatography (HPLC) column (Phenomenex Jupiter, Torrance, CA, USA; 250 × 10 mm, 10 µm particle diameter). The solvents were 0.1% TFA in water (solvent A) and 90% CH_3_CN with 0.1% TFA in water (solvent B). Venom peptides were eluted from the column with a linear gradient ranging from 6% to 60% solvent B at 0.9% solvent B/min, followed by 60% to 100% at 1.3% solvent B/min. The flow rate was 3 mL/min and fractions were collected by peak picking following the absorbance monitored at 220 and 280 nm. Subsequent fractionation of the bioactive fraction was done using an analytical C_18_ HPLC column (Phenomenex Jupiter; 250 × 4.6 mm, 5 µm particle diameter) with a shallower linear gradient ranging from 23% to 27% solvent B at 0.15% solvent B/min at 1 mL/min flow rate, until a single symmetrical peak was achieved.

### 4.3. Peptide Characterization

Mass determination of the native peptides was accomplished by Electrospray Ionization (ESI) Mass Spectrometry (MS) using Waters G2-XS quadrupole—time of flight (q-ToF) at the Institute of Chemistry, University of the Philippines Diliman. Briefly, purified peptide fractions were injected into a Waters 1.7 μm C_18_ column (50 × 2.1 mm) at a flow rate of 500 μL/min and eluted using a 5-min gradient from 5% to 95% B (solvent A: 100% water with 0.1% formic acid; solvent B: 100% CH_3_CN with 0.1% formic Acid). Data were acquired using the Fast Data Dependent Acquisition (FastDDA) method with a selected mass range of 400–2000 *m*/*z* and a survey scan time of 0.4 s.

Mass determination of the native peptides was accomplished by Electrospray Ionization (ESI) Mass Spectrometry (MS) using Waters G2-XS quadrupole—time of flight (q-ToF) at the Institute of Chemistry, University of the Philippines Diliman. Briefly, purified peptide fractions were injected into a Waters 1.7 μm C_18_ column (50 × 2.1 mm) at a flow rate of 500 μL/min and eluted using a 5-min gradient from 5% to 95% B (solvent A: 100% water with 0.1% formic acid; solvent B: 100% CH_3_CN with 0.1% formic Acid). Data were acquired using the Fast Data Dependent Acquisition (FastDDA) method with a selected mass range of 400–2000 *m*/*z* and a survey scan time of 0.4 s.

The amino acid sequence was determined by automated Edman degradation using Shimadzu PPSQ-31B Protein Sequencer. Purified peptide fractions were loaded on a glass fiber disk pre-treated with Sequa-brene™. Phenylisothiocyanate (PITC) was coupled to the amino group at the N-terminus of the peptide in alkaline condition, producing a phenylthiocarbamyl (PTC) peptide. Trifluoroacetic acid was used to cleave the peptide bond at the end of the PTC-peptide, producing the N-terminal amino acid 2-anilino-5-thiazolinone (ATZ). The ATZ-amino acid was extracted with organic solvent and converted to the more stable phenylthiohydantoin (PTH) derivative, and then separated using reverse-phase HPLC. These reactions were repeated for multiple cycles until all amino acids of the peptide were cleaved off. In each cycle, an unknown amino acid was identified by comparing HPLC retention times to a previously analyzed PTH-amino acid standard.

### 4.4. Intracranial Mouse Bioassay

Aliquots of peptide fractions were dried, resuspended in 12 µL normal saline solution (NSS; 0.9% NaCl), and injected intracranially into 14- to 17-day-old mice using a 29-gauge insulin syringe. Negative control mice were injected with NSS only. Mouse behavior after injection was observed for at least three hours. This protocol is approved by the Institutional Animal Care and Use Committee of the University of the Philippines Diliman (MSI-2014-01; approved 8 August 2014) and the University of Utah (17-07020; approved 1 August 2017)

### 4.5. RNA Sequencing and Transcriptome Library Assembly

For whole transcriptome sequencing, total RNA extraction from venom glands of two specimens of *Clavus davidgilmouri* was performed using the Direct-zol RNA extraction kit (Zymo Research, Irvine, CA, USA), with on-column DNase treatment, according to the manufacturer’s instructions. Transcriptome library preparation and sequencing were performed by the University of Utah High Throughput Genomics Core Facility as previously described [24]. The raw reads data are available as bioproject PRJNA610292. The analysis of transcriptomic data was carried out as described by Lu et al. [7].

### 4.6. Putative Toxin Identification

Sequences from transcriptome were identified as potential toxin precursor peptides based on the presence of a characteristic signal sequence, propeptide and toxin regions using website-based computational servers SignalP 5.0 [25], Conoserver [26], and ProP 1.0 [27], classified into gene superfamilies based on conserved sequences of the signal regions and further characterized by cysteine framework. Putative toxin precursors were aligned with MUSCLE using MacVector 17.0 sequence analysis software (MacVector, Inc., PO Box 1147, Apex, NC, USA) and refined by eye. The sequences described here have been deposited into GenBank (Table S1).

### 4.7. Peptide Synthesis

Solid phase Fmoc peptide chemistry was used to generate cdg14a, cdg14 (3–9,15–20), cdg14 (3–15,9–20) and cdg14 (3–20,9–15) with an AAPPTec Apex 396 automated peptide synthesizer (Louisville, KY, USA). The peptide, along with three additional analogs designed to establish disulfide connectivity of the native peptide, was initially constructed on a preloaded Fmoc-Rink Amide MBHA resin (substitution: 0.45 mmol/g; Peptides International Inc., Louisville, KY, USA). All standard amino acids were purchased from AAPPTec. Side-chain protection occurred for the following amino acids: Ser, Thr and Tyr: O-tert-butyl, Cys: trityl (Trt), and Asp: tert-butyl. For cdg14 (3–9,15–20), Cys3 and Cys9 were protected with an acetamidomethyl (Acm) group and Cys15 and Cys20 with a trityl group; for cdg14 (3–15,9–20) Cys3 and Cys15 were Acm protected while Cys9 and Cys15 were Trt protected; whereas in cdg14 (3–20,9–15) Cys3 and Cys20 were Acm protected, and Cys9 and Cys15 were Trt protected. The peptides were synthesized at 50 μmol scale. Coupling activation was achieved with 1 equivalent of 0.4 M benzotriazol-1-yl- oxytripyrrolidinophosphonium hexafluorophosphate and 2 equivalents of 2 M *N*,*N*-diisopropylethyl amine in N-methyl-2-pyrrolidone as the solvent. For each coupling reaction, a 10-fold excess of amino acid was used, and the reaction was carried out for 60 min. Fmoc deprotection was performed for 20 min with 20% (*v*/*v*) piperidine in dimethylformamide. 

Peptide cleavage and purification were performed in the following manner. Linear cdg14a was cleaved from 108 mg resin by a 2.5-h treatment with 1 mL of Reagent K (TFA/water/phenol/thioanisole/1,2-ethanedithiol 82.5/5/5/5/2.5 by volume). Next, the cleavage mixture was filtered and precipitated with 10 mL of cold methyl-tert-butyl ether (MTBE). The crude peptide was then precipitated by centrifugation at 7000× *g* for 6 min and washed once with 10 mL cold MTBE. Because of the poor solubility of the crude linear peptide, it was first treated with positively charged 2-Aminoethyl methanethiosulfonate hydrobromide (MTSEA; Biotium, Fremont, CA, USA) in order to temporarily modify all cysteines and increase peptide hydrophilicity. Crude peptide was re-suspended in 50% CH_3_CN in 0.1% TFA in water; then 50 mg of MTSEA was added. The reaction mixture was vortexed and left for 1 h at room temperature. It was then diluted with 0.1% TFA in water and purified by reversed-phase (RP) HPLC using a semi-preparative C4 Vydac column (204TP510, 250 × 10 mm, 5-μm particle size) eluted with a linear gradient ranging from 15% to 45% solvent B in 30 min at a flow rate 4 mL/min. The HPLC solvents were 0.1% (*v*/*v*) TFA in water (solvent A) and 0.1% TFA (*v*/*v*) in 90% aqueous CH_3_CN (*v*/*v*) (solvent B). The eluent was monitored by measuring absorbance at 220 and 280 nm. The purity of the peptide was assessed by analytical C_18_ Vydac RP-HPLC (218TP54, 250 × 4.6 mm, 5-μm particle size) using the same gradient as described above with a flow rate of 1 mL/min. The peptide was quantified by UV absorbance at 280 nm, using an extinction coefficient (ε) value of 4470 M^−1^·cm^−1^. The identity of the peptide was confirmed by ESI-MSI (calculated (M) = 2574.92 Da; determined (M) = 2574.92 Da) at the Mass Spectrometry and Proteomics Core Facility at the University of Utah.

The same method of linear peptide modification was used for cdg14 (3–9,15–20), cdg14 (3–15,9–20) and cdg14 (3–20,9–15) The bis-aminoethanethiol modified peptides were obtained on average in 62–85% purity, and identity was confirmed by ESI MS. The mass found was (M) = 2566.98 Da (calculated (M) = 2566.99 Da).

### 4.8. Oxidative Folding of cdg14a in the Presence of Oxidized and Reduced Glutathione

Two hundred nanomoles of linear cdg14a was resuspended in 1 mL of 50% CH_3_CN in 0.01% TFA solution and added to a solution containing the following: 5 mL of 0.2 M Tris-HCl (pH = 7.5) plus 0.2 mM EDTA, 0.5 mL of 20 mM reduced and 0.5 mL of 20 mM oxidized glutathione, and 3 mL of water. Final peptide concentration in the folding mixture was 20 µM. The folding reaction was conducted for 24 h to obtain the native-like peptide cdg14a and 30 min to obtain the more hydrophobic, kinetically favorable form cdg14a-pk2, then quenched with formic acid to a final concentration of 8%. The quenched reaction mixture was then separated by RP-HPLC using a semi-preparative C_18_ column and a linear gradient ranging from 15% to 45% of solvent B in 30 min with a flow rate of 4 mL/min. The eluent was monitored by absorbance at 220 and 280 nm. The purity of the folded peptide was assessed by an analytical C_18_ Vydac RP-HPLC using the gradient described for the linear peptide, with a flow rate of 1 mL/min. Pure, fully folded cdg14a and cdg14a-pk2 were quantified by absorbance at 280 nm as described for the linear peptide. Out of 2000 nmols of linear peptide, 880 nmols of cdg14a were obtained with 98% purity (44% yield), and out of 745 nmols, 192 nmols of the more hydrophobic isomer, cgd14a_pk2, were produced with 95% purity (26% yield). The molecular mass of cdg14a and cdg14a_pk2 was confirmed by MALDI MS at Mass Spectrometry and Proteomics Core Facility at the University of Utah. For cdg14a, the major mass determined was a sodium adduct 2293.92 Da, where the expected mass (M + H)^+^ = 2271.91 Da was a minor signal (calculated (M + H)^+^ = 2271.84 Da). For cdg14a_pk2, an additional potassium adduct was also observed with a mass of 2309.87 Da.

The same method of oxidative folding was used to close the first bridge in cdg14 (3–9,15–20), cdg14 (3–15,9–20) and cdg14 (3–20,9–15) with the average yield of 46% and purity ranging between 87% and 92%. The identity of the peptides was confirmed by MALDI MS. The major mass observed was identified to be a sodium adduct (M+ Na)^+^ = 2438.02 Da (calculated (M + Na)^+^ = 2437.92 Da) and the minor to be a potassium adduct for all 3 folding isomers (M + K)^+^ = 2453.89 Da (calculated (M + K)^+^ = 2454.02 Da).

Oxidative folding of cdg14 (3–9,15–20), cdg14 (3–15,9–20) and cdg14 (3–20,9–15) was performed in the presence of iodine. In brief, 10 mg of iodine was dissolved in 5 mL of CH_3_CN, and 15 mL of water and 0.6 mL of TFA were added. A total of 168 nmol of monocyclic, cdg14 (3–15,9–20) in 5 mL of HPLC solvents was added to 5 mL of the iodine solution, and the reaction was stirred for 10 min. Then it was quenched with 100 µL of freshly prepared 1 M ascorbic acid in water. The reaction was diluted to 16 mL with solvent A and purified by RP-HPLC using a semi-preparative C_18_ column and a linear gradient ranging from 15% to 45% of solvent B in 30 min with a flow rate of 4 mL/min. Pure, fully folded cdg14 (3–15,9–20) was quantified by absorbance at 280 nm as described for the linear peptide to yield 112 nmol of the fully folded peptide (yield: 65%, purity: 97%; t_R_ = 16.07 min). The identity of the peptide was confirmed by MALDI MS. The major mass determined was a sodium adduct (M + Na)^+^ = 2293.93 Da (calculated: 2293.82) and potassium adduct (M + K)^+^ = 2309.91 Da (calculated: 2309.88 Da), where the expected mass (M + H)^+^ = 2271.93 Da was a minor signal (calculated (M + H)^+^ = 2271.84). The same protocol was applied to prepare the bead analog cdg14 (3–9,15–20) (yield: 82%; purity: 95% t_R_ = 22.92 min) and ribbon analog cdg14 (3–20,9–15)(yield: ~20% (due to precipitation); purity: 95%; t_R_ = 20.51 min).

### 4.9. Co-Elution of the Native and Synthetic cdg14a

To determine whether the native and synthetic cgd14a peptides were identical, both peptides were loaded separately on a C_18_ analytical column with a linear gradient of 20–23% of solvent B in 20 min at 1 mL/min to compare their retention times. A co-elution experiment was also done by mixing native and synthetic cdg14a peptides in a 1:2 ratio and loaded on a C_18_ analytical column using the same gradient.

### 4.10. Constellation Pharmacology

Experiments involving the use of live animals were approved by the Institutional Animal Care and Use Committee (IACUC) of the University of Utah (17-05017; approved 31 May 2017). Dorsal root ganglia (DRG) were dissected from 6- to 8-week old CD1 mice engineered to express the green fluorescent protein (GFP) under the control of the calcitonin gene related peptide (CGRP) promoter, thus GFP was used for labeling CGRP-positive cells. DRGs were dissociated, plated on poly D-lysine-coated 24-well plates, and cultured overnight in minimum essential media (MEM) containing 10% FBS, 100 U/mL penicillin, 100 μg/mL streptomycin, 1× glutamax, 0.4% (*w*/*v*) glucose, and 10 mM HEPES in a humidified incubator at 37 °C and 5% CO_2_. The cells were loaded with FURA-2-AM dye one hour before the experiment. The cells were then washed 3× with DRG observation solution (145 mM NaCl, 5 mM KCl, 2 mM CaCl_2_, 1 mM MgCl_2_, 1 mM Na-Citrate, 10 mM HEPES, 10 mM glucose and 1× Pen-Strep) and allowed to recover for 10 min before the experiment was started. The ability of cdg14a to modulate calcium transients in response to the application of 20 mM K^+^ was measured. Three pulses of 20 mM K^+^ each for 10 s were applied to the DRG neurons at 6-min intervals. The neurons were washed with DRG observation solution 3× after each K^+^ pulse. After the third K^+^ pulse, the neurons were incubated with 10 nM cdg14a for 5 min; then a solution containing 20 mM K^+^ plus 10 nM cdg14a was applied to the cells for 10 s. The cells were again washed 3× with a DRG observation solution and incubated with 100 nM cdg14a for 5 min. After 5 min, a solution of 20 mM K^+^ plus 100 nM cdg14a was again applied to the neurons for 10 s. The cells were allowed to recover for 12 min, and then 20 mM K+ was applied to determine the reversibility of the cdg14a effect. The cells were then incubated for 5 min in a DRG observation solution containing 1 μM of the conopeptide κM-RIIIJ to identify cells expressing Kv1.1 and/or Kv1.2 [28]. Direct response to κM-RIIIJ, as well as response to 20 mM K+ after κM-RIIIJ incubation, indicating an indirect response, characterizes classes of DRG neurons [4]. The cells were then incubated for 15 s at 6-min intervals with the following: AITC (100 μM), menthol (400 μM), capsaicin (300 nM), and K+ (40 mM). IB4 and HOECHST staining were performed after calcium imaging experiments. Then a GFP image to visualize CGRP positive cells, a Cy5 image to visualize IB4 positive cells, and a bright-field image were taken after the calcium imaging experiment. Responses to the challenge compounds and the characteristic IB4 and CGRP labeling were used to group cells into different subclasses [20]. Estimates of amplification resulting from 10 nM cdg14a application are reported as mean ± SE. Means are computed as % maximal response to 20 mM K^+^ in the presence of cdg14a relative to 20 mM K^+^ response in the absence of the peptide.

## Figures and Tables

**Table 1:** Effects of cdg14a on mice ^b^.

Average Weight ± Deviation (g)	nmol of cdg14a in NSS	Observations (Intracranial Injection)
12.1 ± 0.1	0	No unusual behavior observed
10.4 ± 0.3	1	Mild shaking at 15 min; frequent scratching and grooming at 22 min lasting 2.5 h.
10.6 ± 0.1	2.5	Shaking at 7 min; frequent scratching and grooming at 11 min; licking of front paw and thumping of hind paw at 1 h lasting 2.5 h. One mouse had convulsions at 3.5 h but recovered.
11.06 ± 0.26	5	Shaking, frequent scratching, and grooming at 7 min; licking of front paw and thumping of hind paw at 18 min lasting 18 min; seizure at 40 min; lying on its side for the rest of the experiment (>3 h).
11.6 ± 0.2	10	Shaking, frequent scratching, and grooming at 5 min; licking of front paw and thumping of hind paw at 9 min lasting 18 min; seizure at 37 min; lying on its side for the rest of the experiment. Mice died at 2 h.

^b^ Swiss Webster mice, n = 2 per dose, male and female; this table reflects data on synthetic cdg14a.

## Supplementary Materials

The following additional materials are available online at https://www.mdpi.com/2072-6651/12/8/508/s1, Table S1: List of GenBank Accession Numbers for CvDg peptide precursor sequences, Table S2: Complete census of subclasses of cells showing a response to cdg14a. Values represent percent of all the cells in each class that respond to cdg14a, Figure S1: Edman sequencing of cdg14a. Reliable reads were obtained only until 21 cycles, Figure S2: Mass spectrum of native cdg14a and calculated mass, Figure S3: Mass spectrum of reduced (dithiothreitol) and alkylated (iodoacetamide) cdg14a and calculated mass, Figure S4: MS/MS fragmentation spectrum of reduced and alkylated cdg14a, Figure S5: Co-elution experiment of the globular and bead forms prepared in one and two step folding. Video S1: Foot thumping phenotype observed in young mice when injected with cdg14a. Video S2: Negative control mouse.
