# Supplementary material for: Purification and Characterization of the Pink-Floyd Drillipeptide, a Bioactive Venom Peptide from Clavus davidgilmouri (Gastropoda: Conoidea: Drilliidae)
Source: Toxins (Basel). 2020 Aug 7;12(8):508. doi: 10.3390/toxins12080508 (PMC7472735; doi:10.3390/toxins12080508)
Supplement: Supplementary file 1 [file toxins-12-00508-s001.zip › toxins-843892-supple proof/toxins-843892 Supplementary Materials.docx]

Supplementary Materials: Purification and Characterization of the Pink-Floyd Drillipeptide, a Bioactive Venom Peptide from *Clavus davidgilmouri* (Gastropoda: Conoidea: Drilliidae)

Victor M. Chua, Joanna Gajewiak, Maren Watkins, Samuel S. Espino, Iris Bea L. Ramiro, Carla A. Omaga, Julita S. Imperial, Louie Paolo D. Carpio, Alexander Fedosov, Helena Safavi-Hemami, Lilibeth A. Salvador-Reyes, Baldomero M. Olivera and Gisela P. Concepcion

**Table S1.** List of Accession Numbers for CvDg peptide precursor sequences.

| **Peptide precursor** | **GenBank Accession Number** |
| --- | --- |
| CvDg14.1 | MK895473 |
| CvDg14.2 | MK895474 |
| CvDg14.2ii | MK895475 |
| CvDg14.3 | MK895476 |
| CvDg14.4 | MK895477 |
| CvDg14.5 | MK895478 |
| CvDg14.14 | MK895479 |
| CvDg14.15 | MK895480 |
| CvDg14.16 | MK895481 |

**Table S2.** Complete census of subclasses of DRG neurons showing a response to cdg14a. Values represent percent of all the cells in each class that respond to cdg14a.

| **Experiment no.** | **L1** | **L2** | **L3** | **L4** | **L5** | **L6** | **G7** | **G8** | **G9** | **G10** | **R11** | **R12** | **R13** | **N14** | **N15** | **N16** |
| --- | --- | --- | --- | --- | --- | --- | --- | --- | --- | --- | --- | --- | --- | --- | --- | --- |
| 1 | 0 | 0 | 13 | 0 | 25 | 0 | 44 | 34 | 37 | 28 | 28 | 0 | 74 | 8 | 41 | 0 |
| 2 | 0 | 0 | 0 | 11 | 0 | 0 | 0 | 46 | 57 | 23 | 26 | 6 | 52 | 7 | 4 | 0 |
| 3 | 0 | 1 | 0 | 0 | 21 | 0 | 25 | 54 | 42 | 40 | 33 | 22 | 66 | 19 | 6 | 0 |
| Average | 0 | 0 | 4 | 4 | 15 | 0 | 23 | 45 | 45 | 30 | 29 | 9 | 64 | 11 | 17 | 0 |


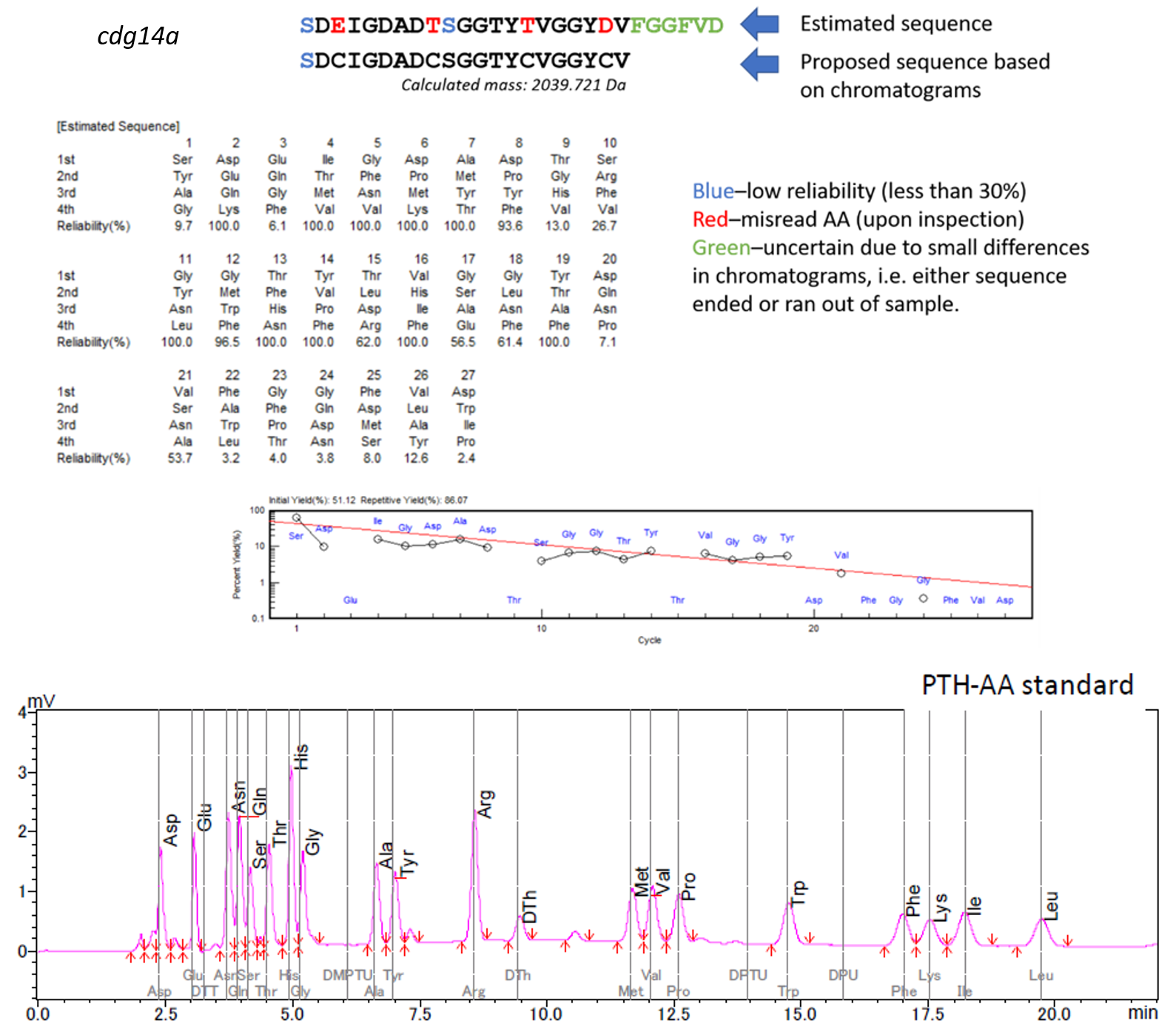


Cycle 1—Ser


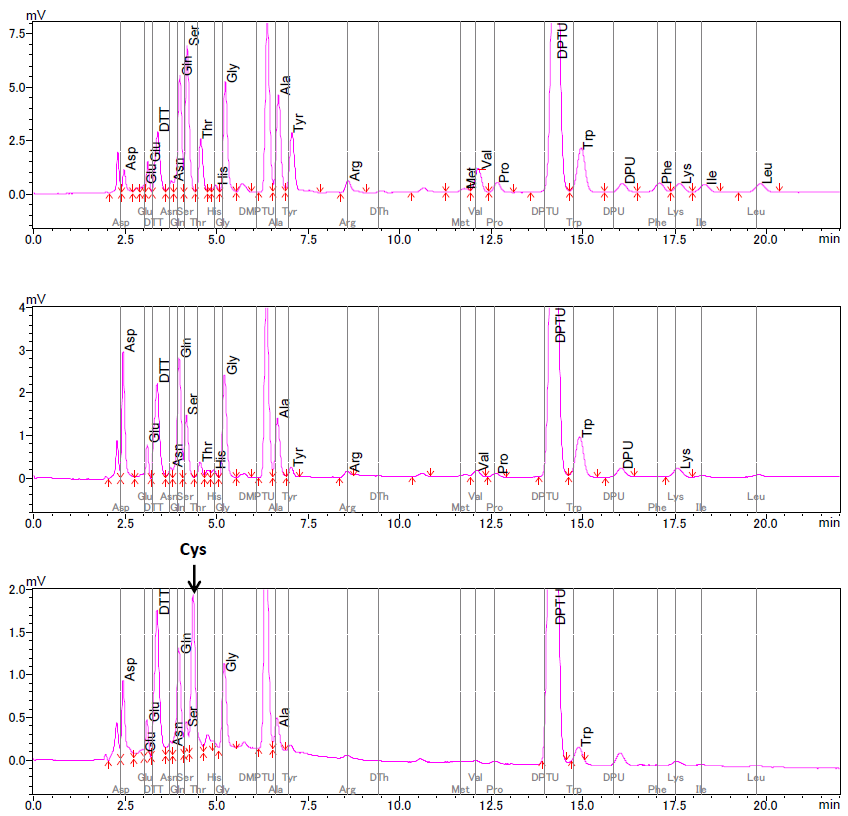


Cycle 2—Asp


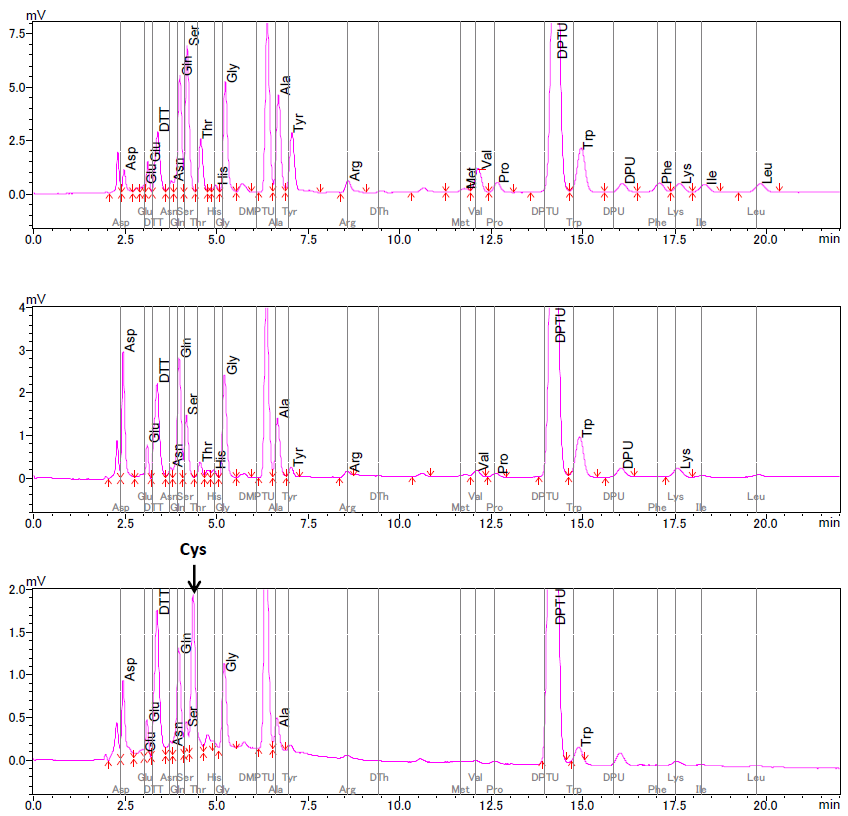


Cycle 3—Glu x


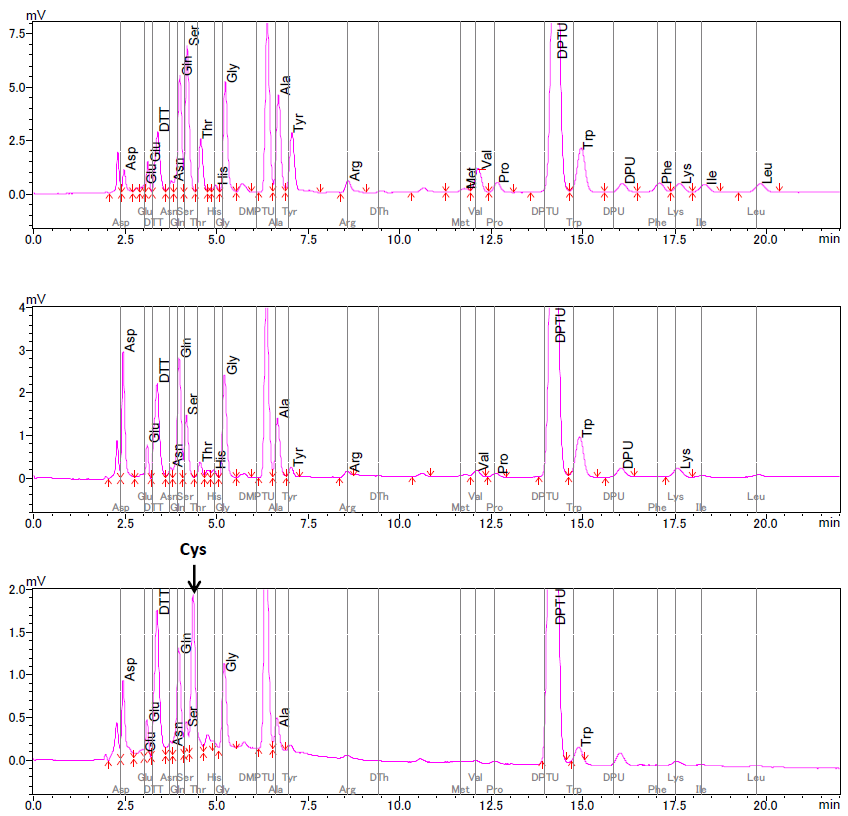


Cycle 4—Ile


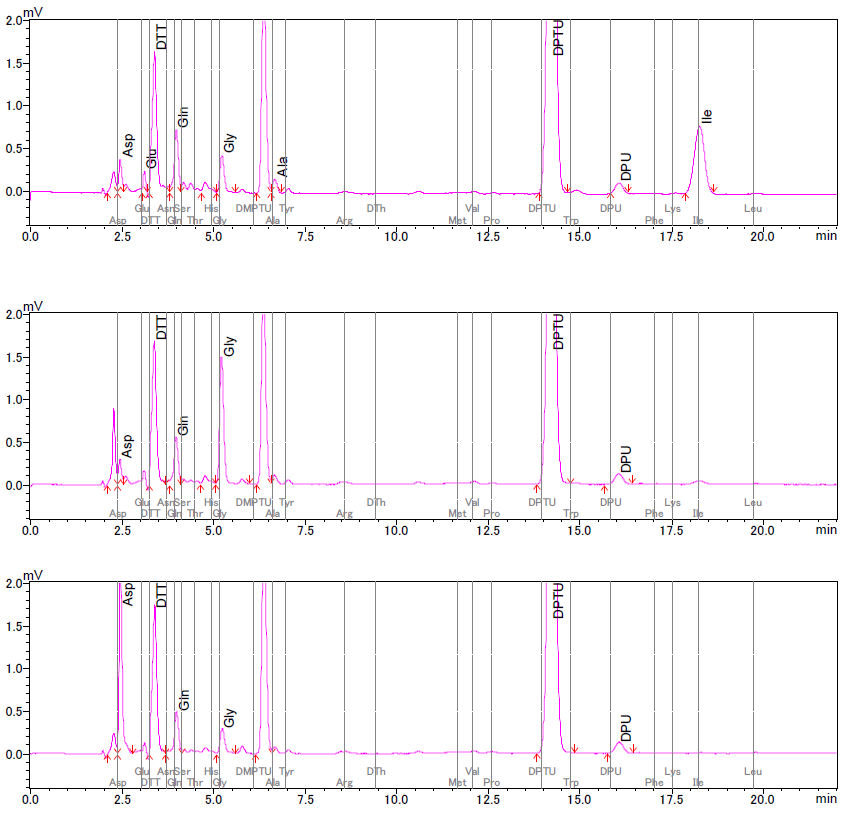


Cycle 5—Gly


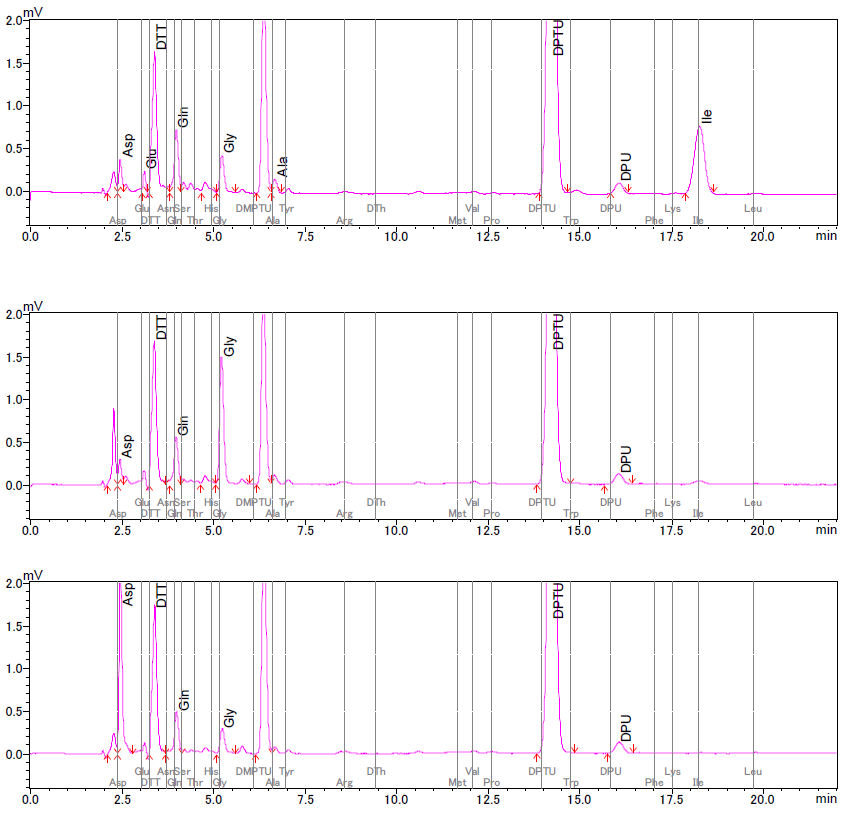


Cycle 6—Asp


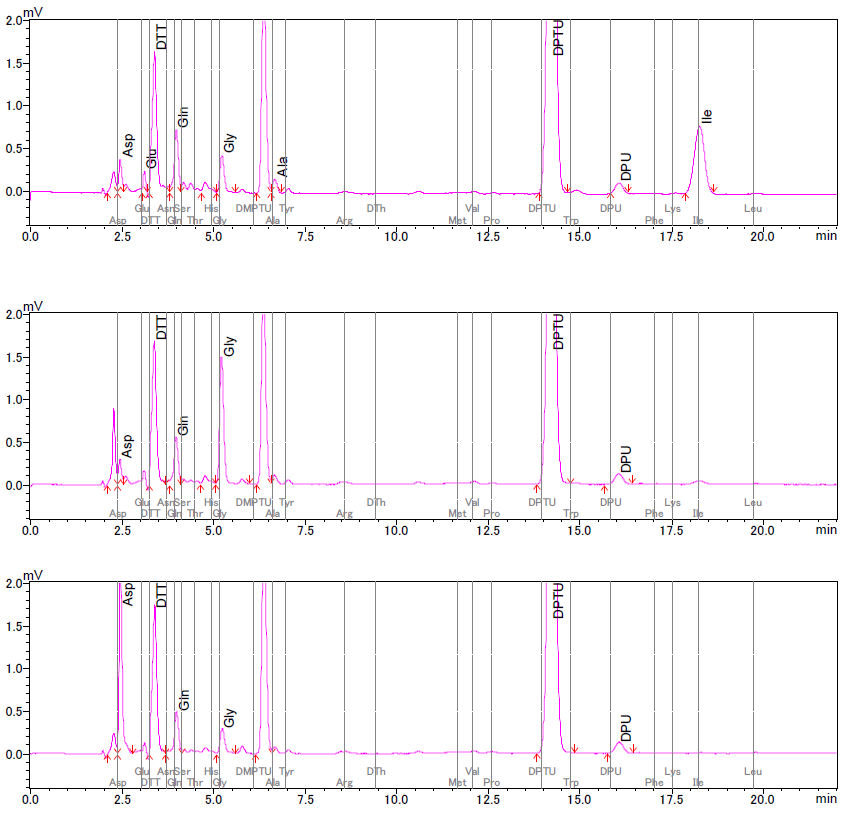


Cycle 7—Ala


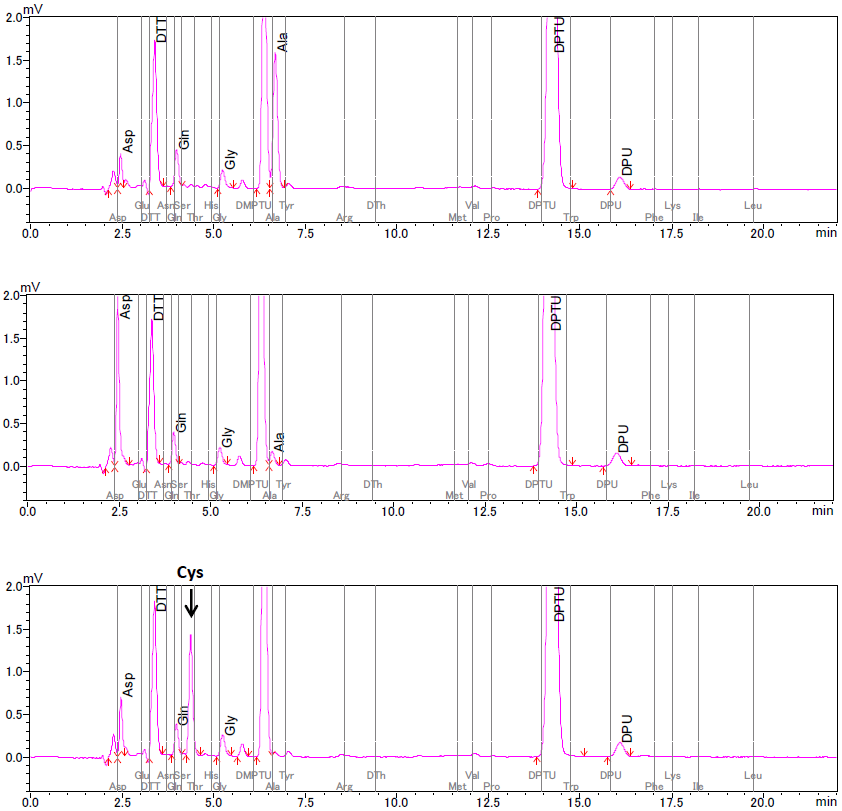


Cycle 8—Asp


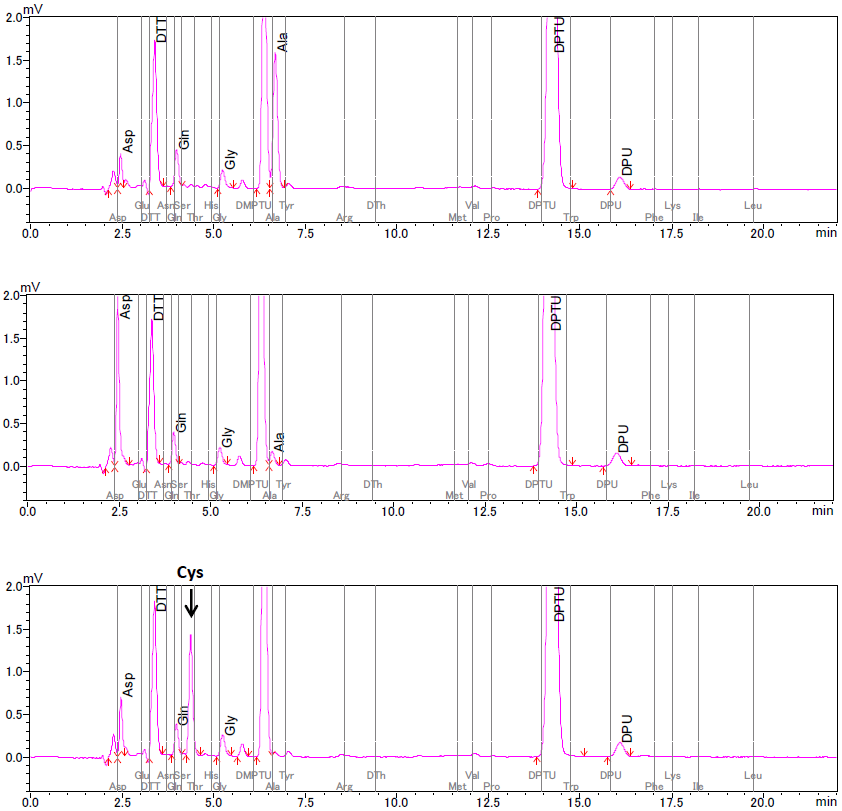


Cycle 9—Thr x


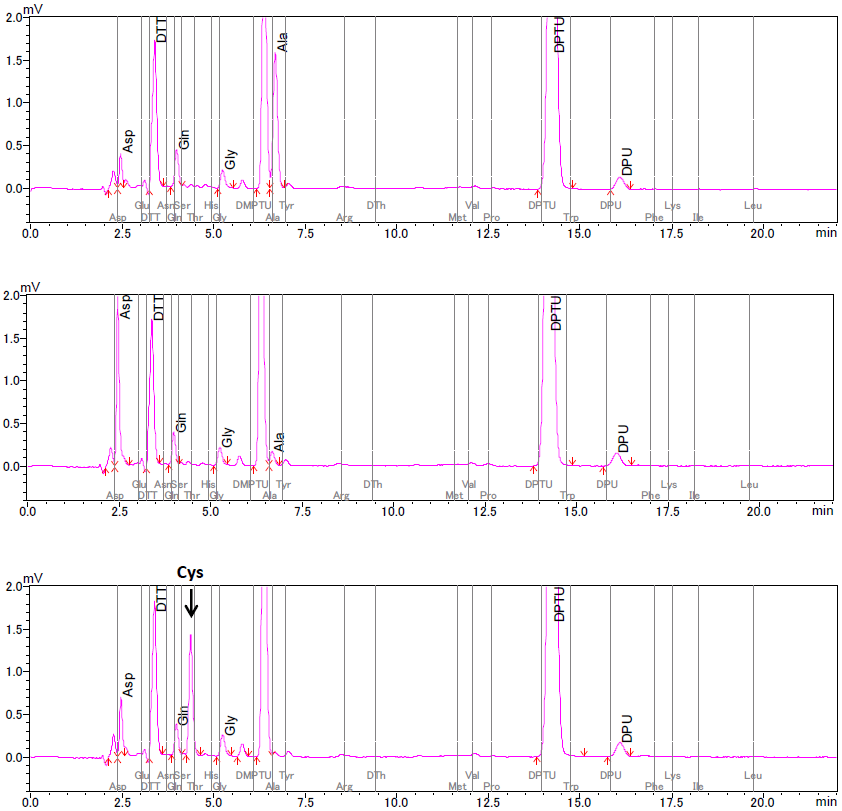


Cycle 10—Ser


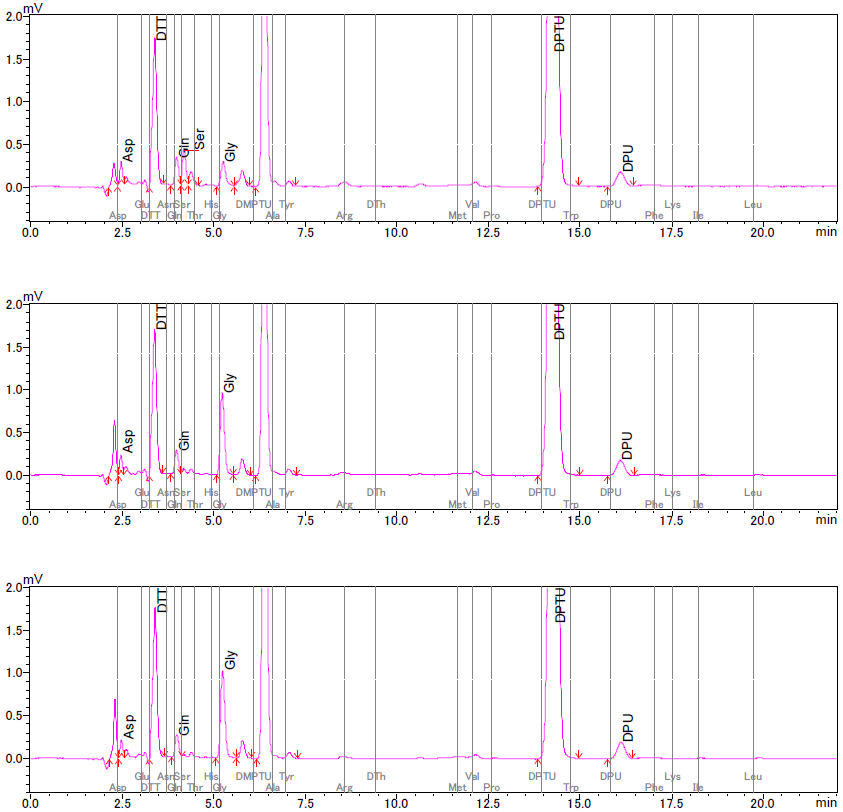


Cycle 11—Gly


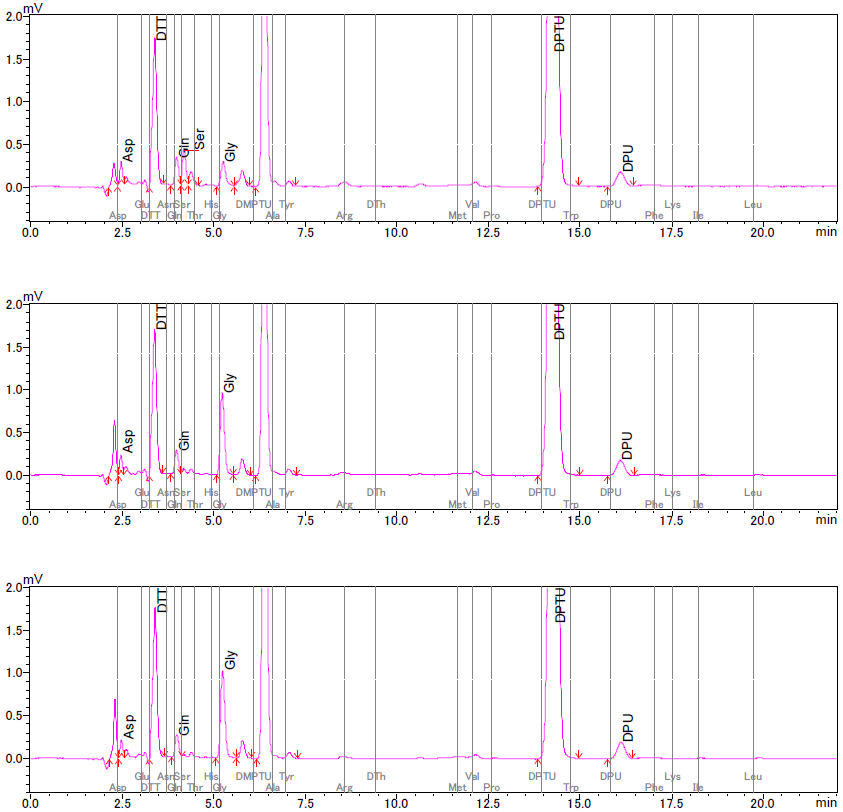


Cycle 12—Gly


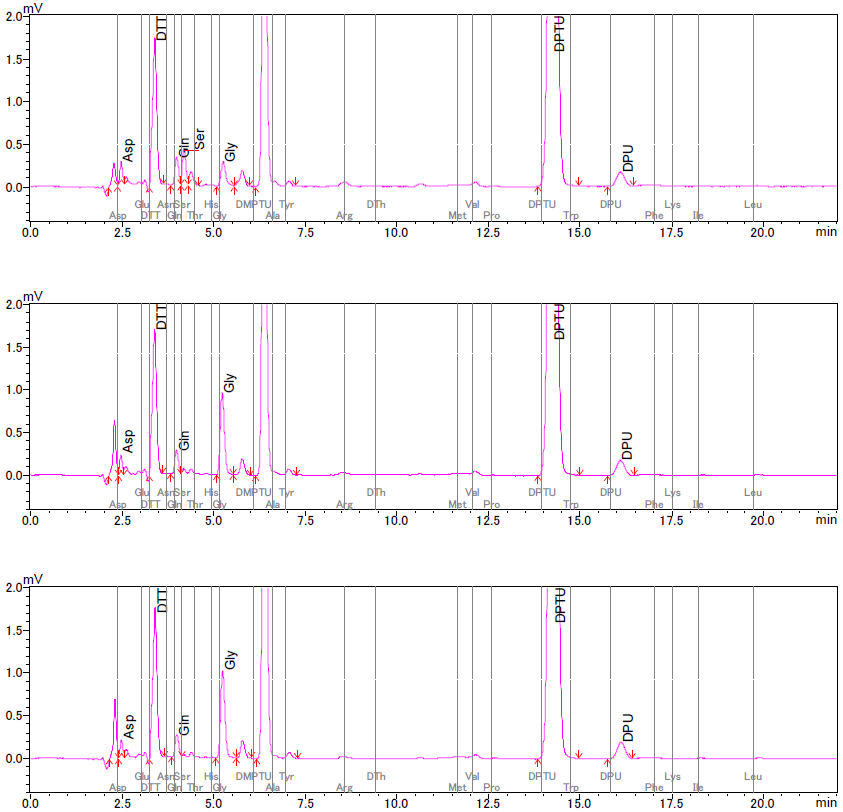


Cycle 13—Thr


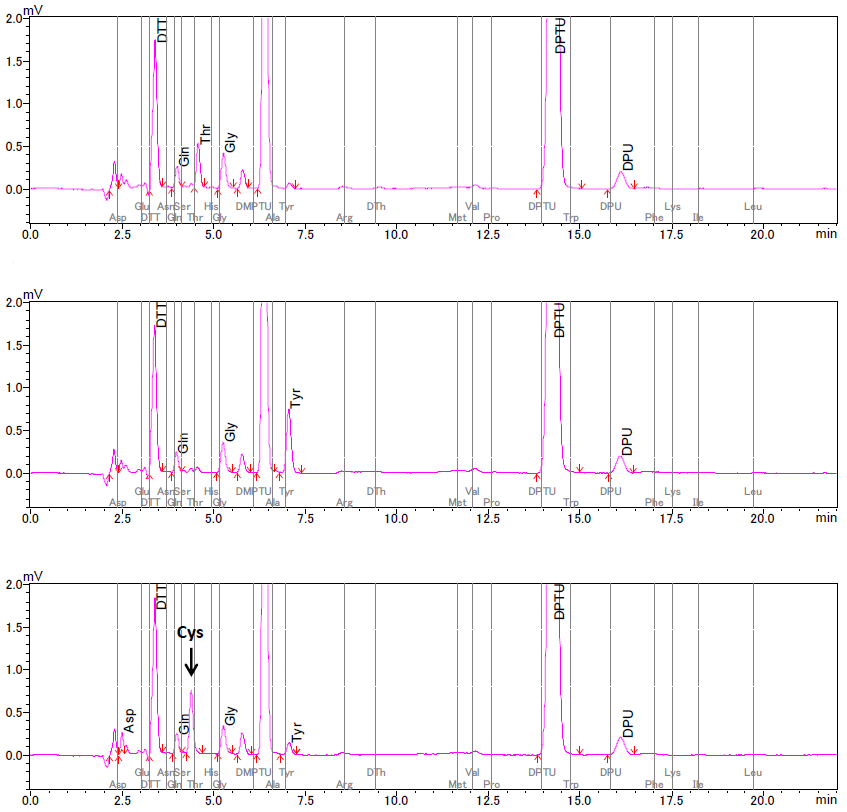


Cycle 14—Tyr


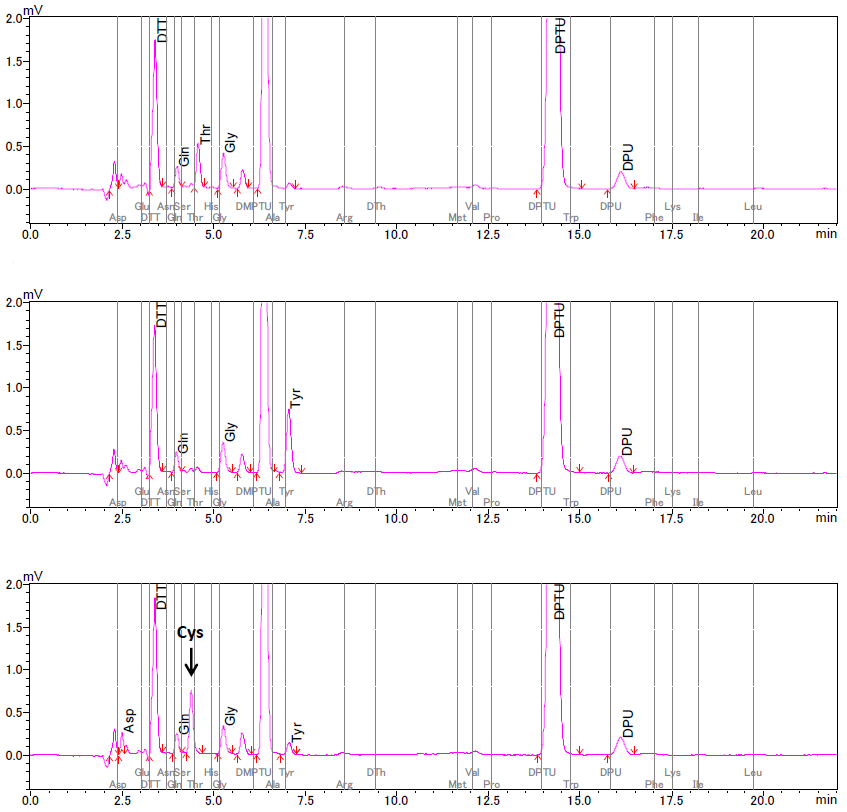


Cycle 15—Thr x


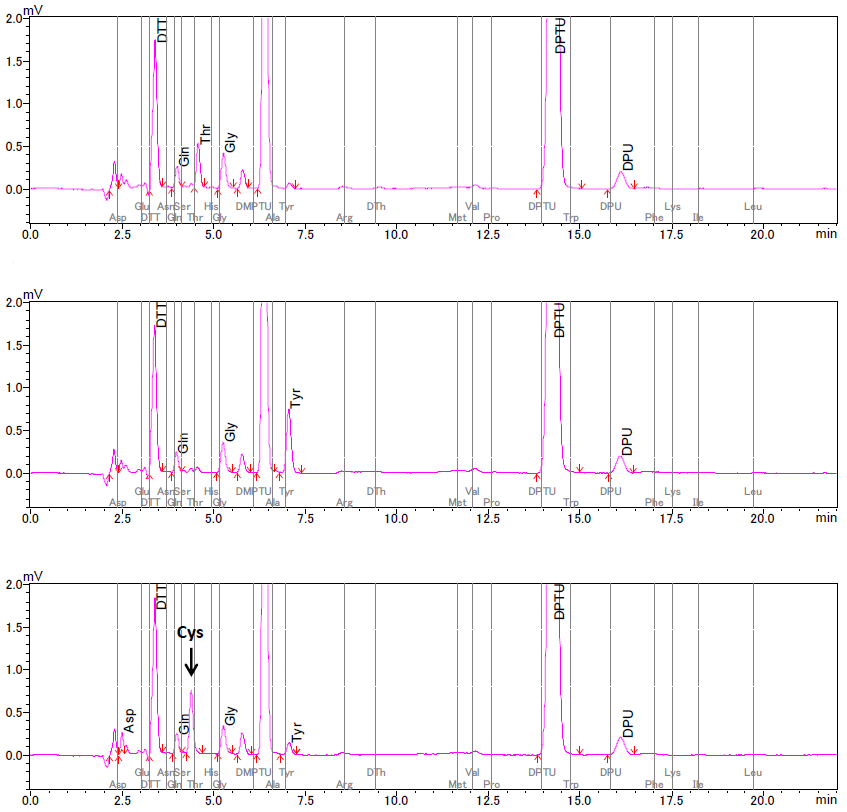


Cycle 16—Val


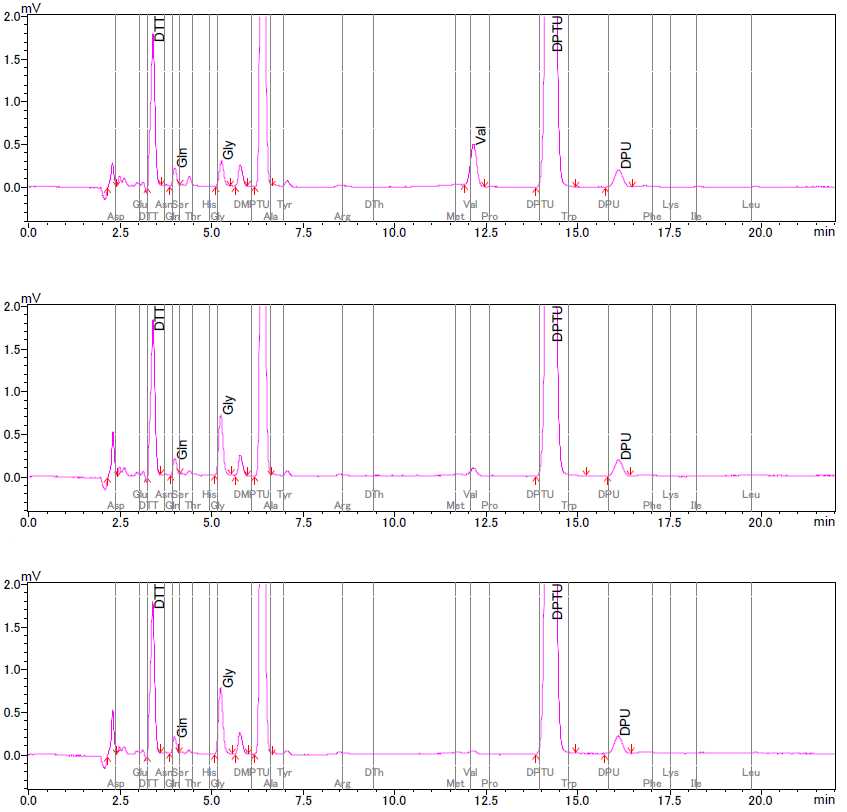


Cycle 17—Gly


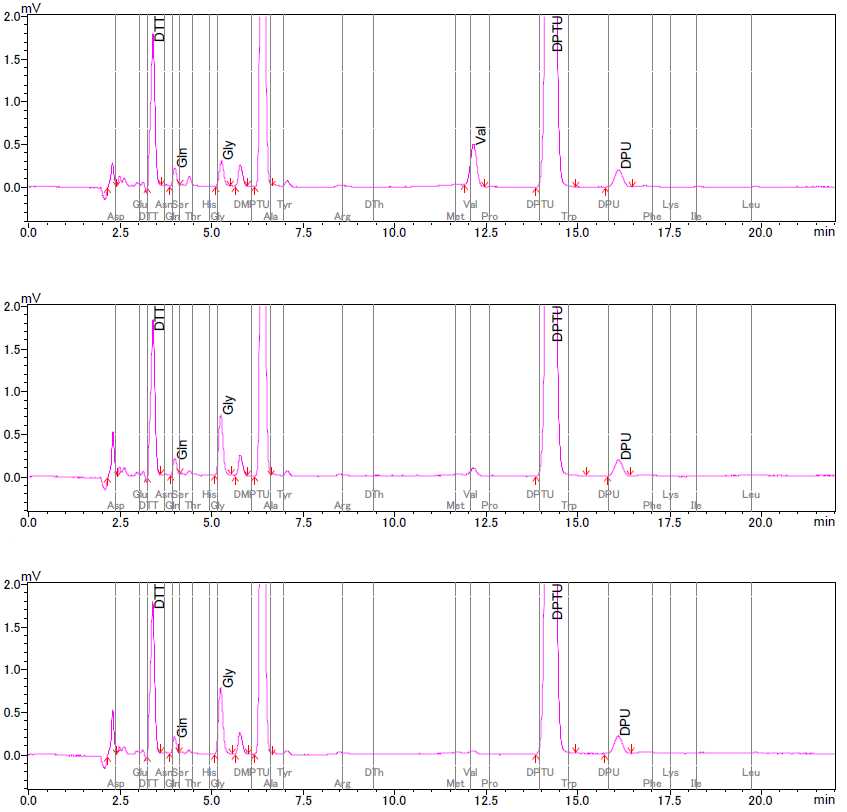


Cycle 18—Gly


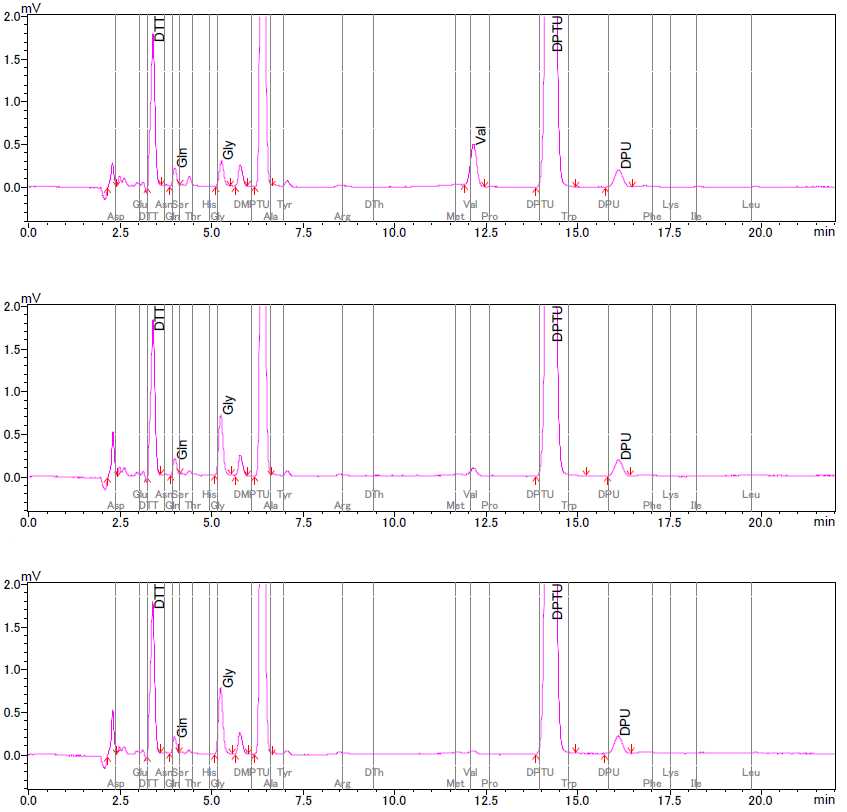


Cycle 19—Tyr


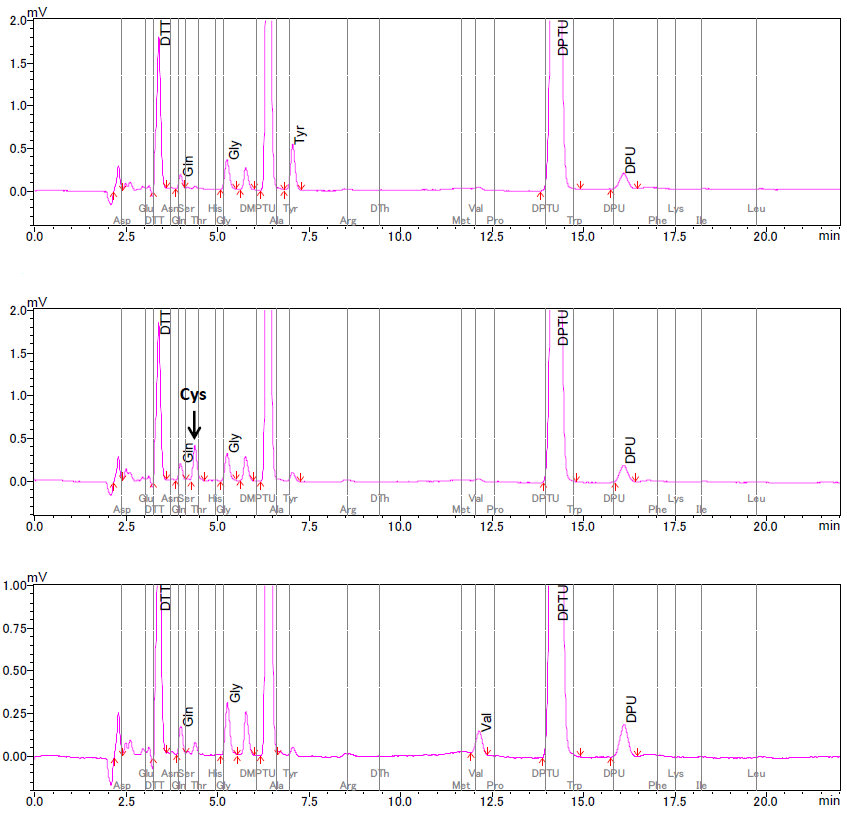


Cycle 20—Asp x


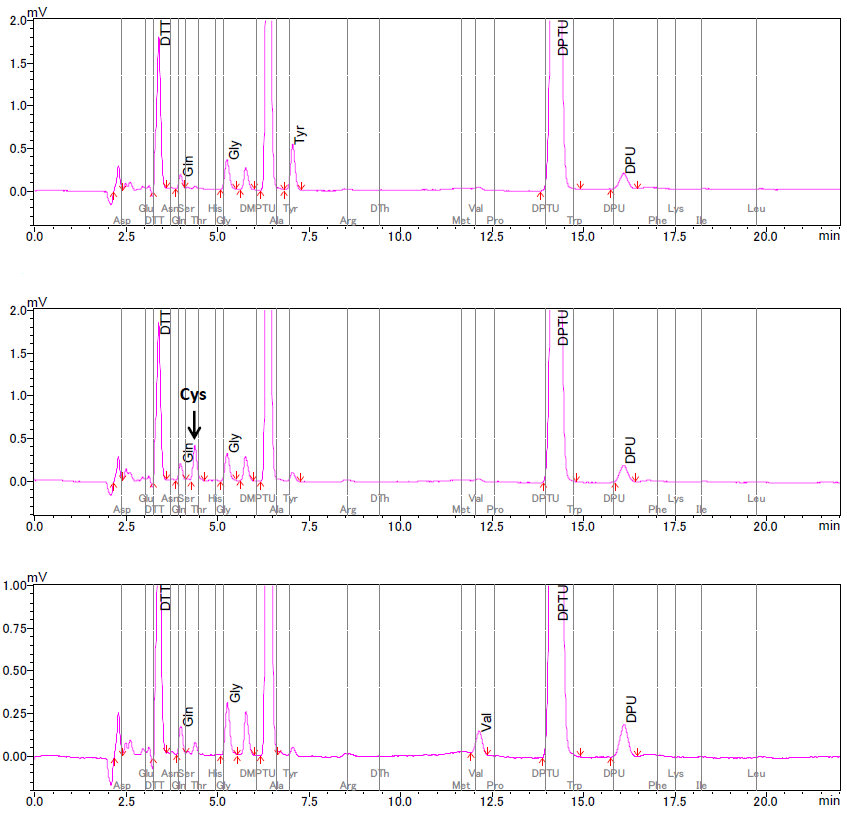


Cycle 21—Val


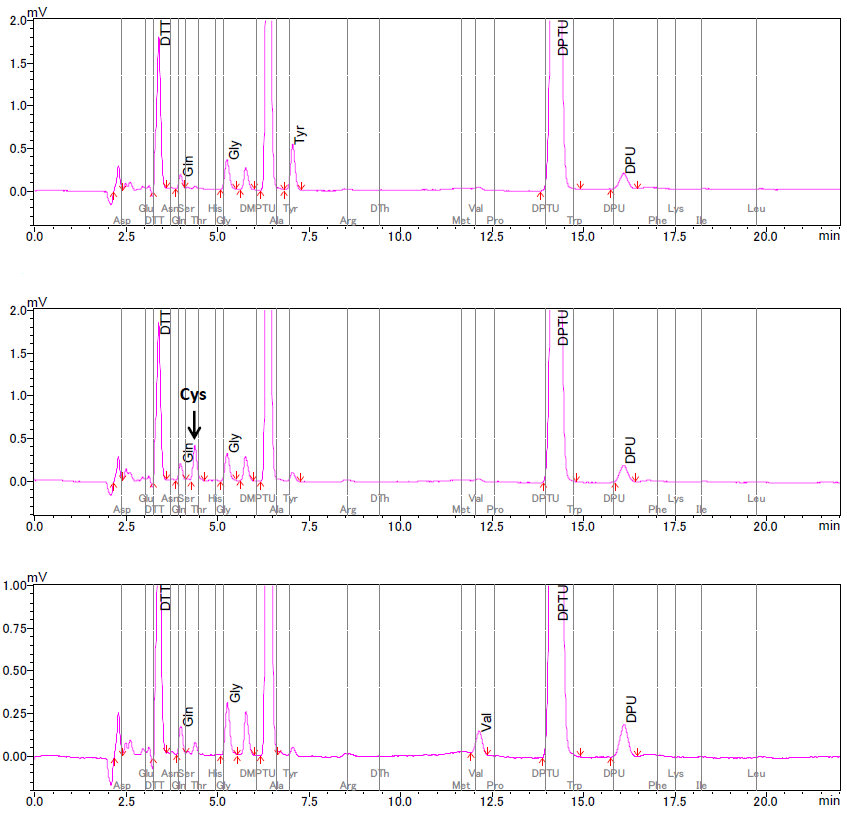


Cycle 22—Phe?


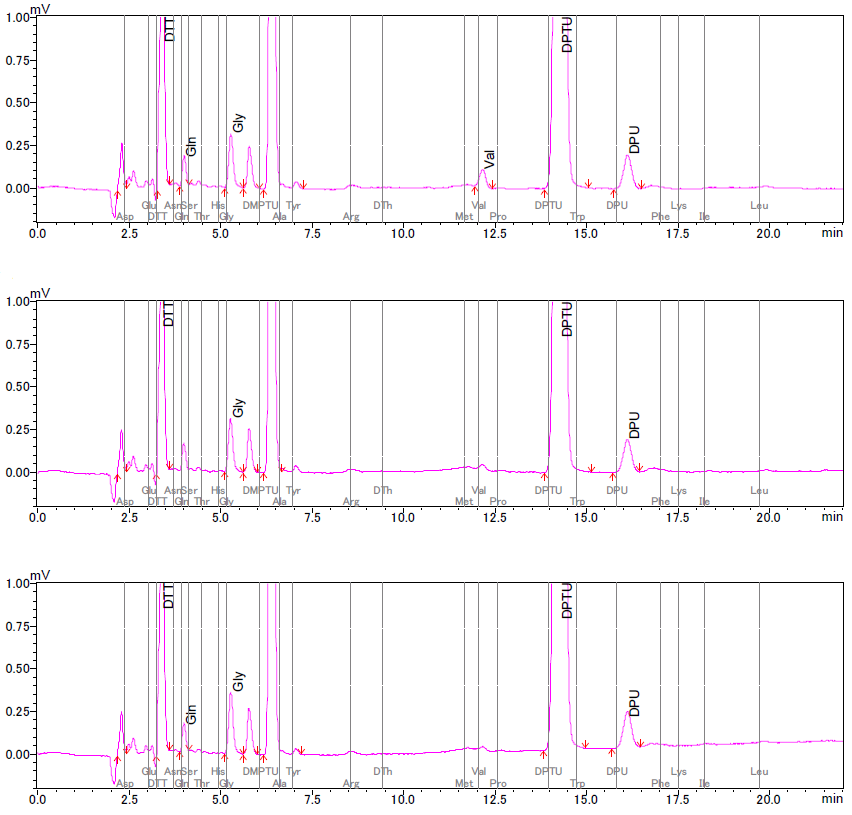


Cycle 23—Gly?


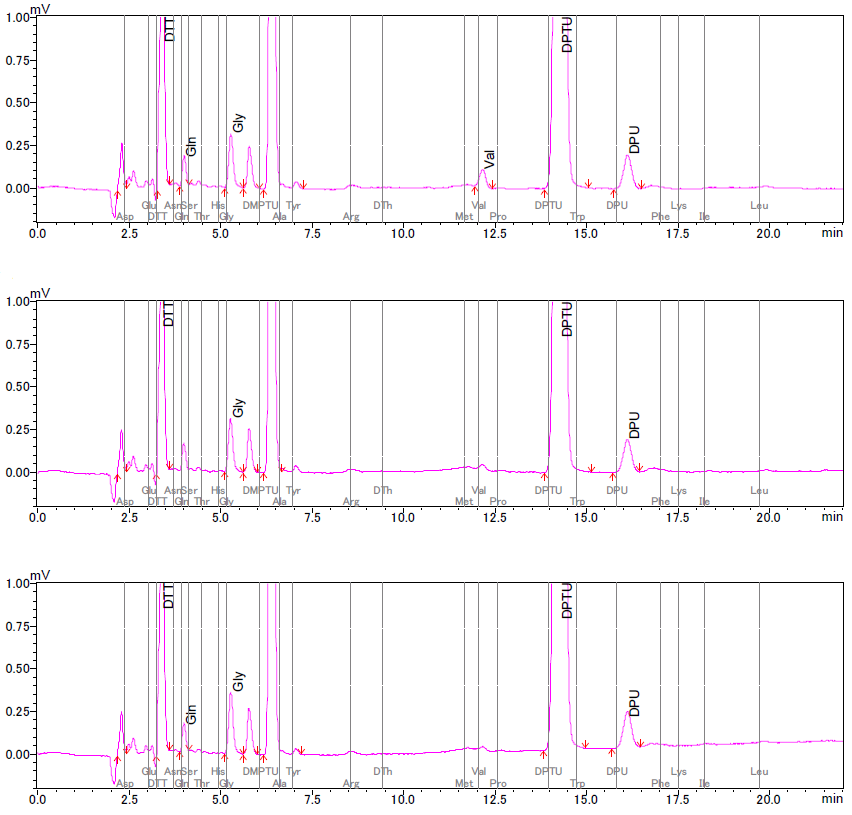


Cycle 24—Gly?


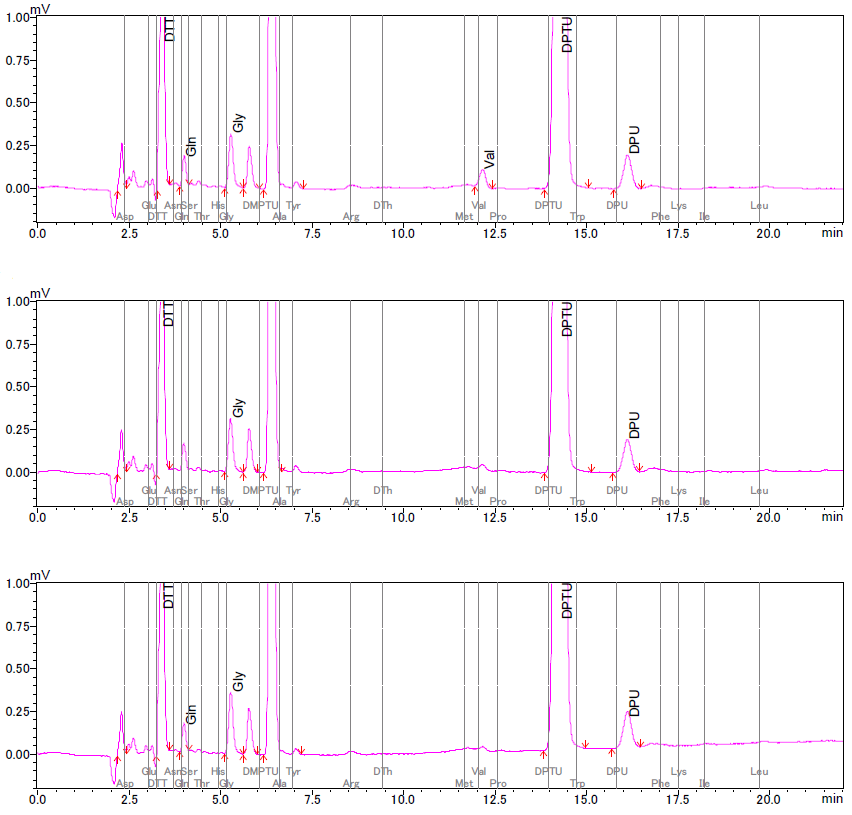


Cycle 25—Phe?


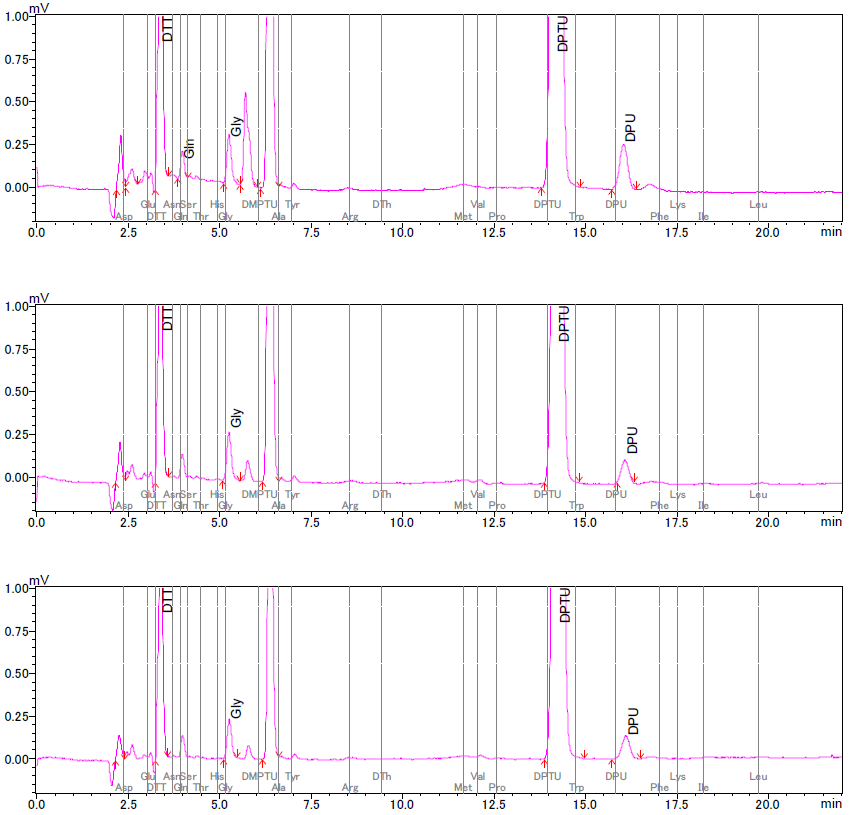


Cycle 26—Val?


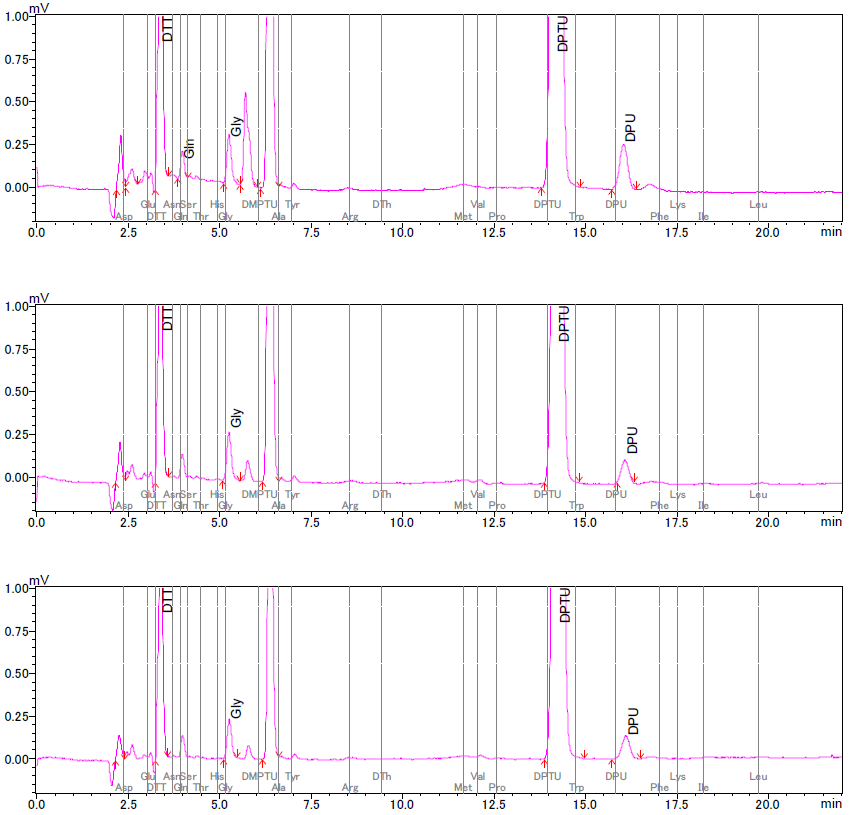


Cycle 27—Asp?


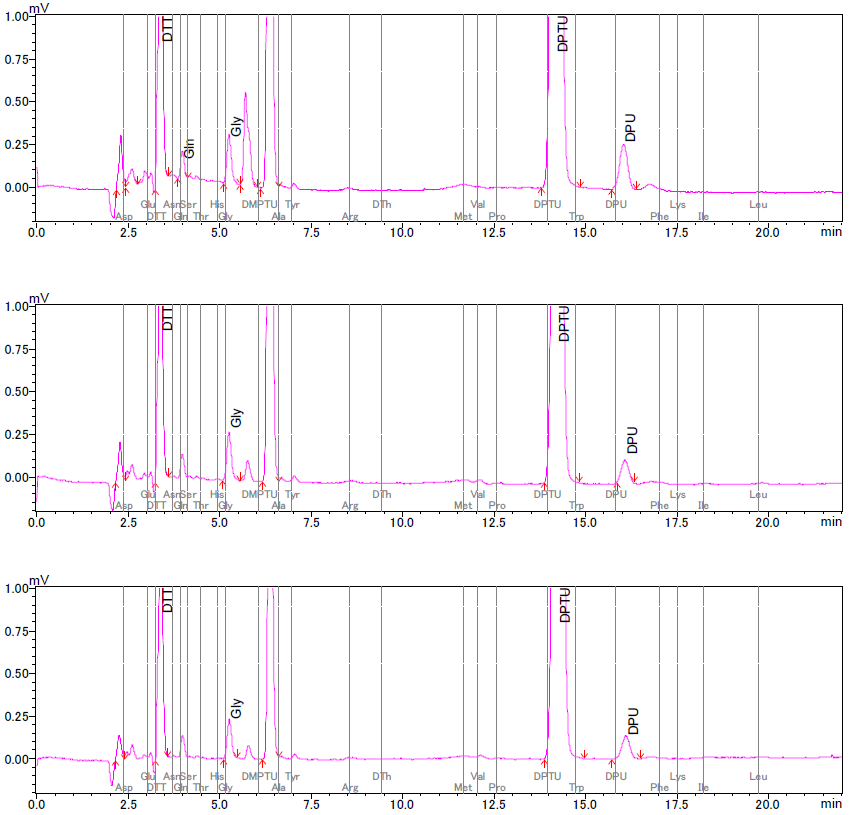


**Figure S1.** Edman sequencing of cdg14a. Reliable reads were obtained only until 21 cycles.


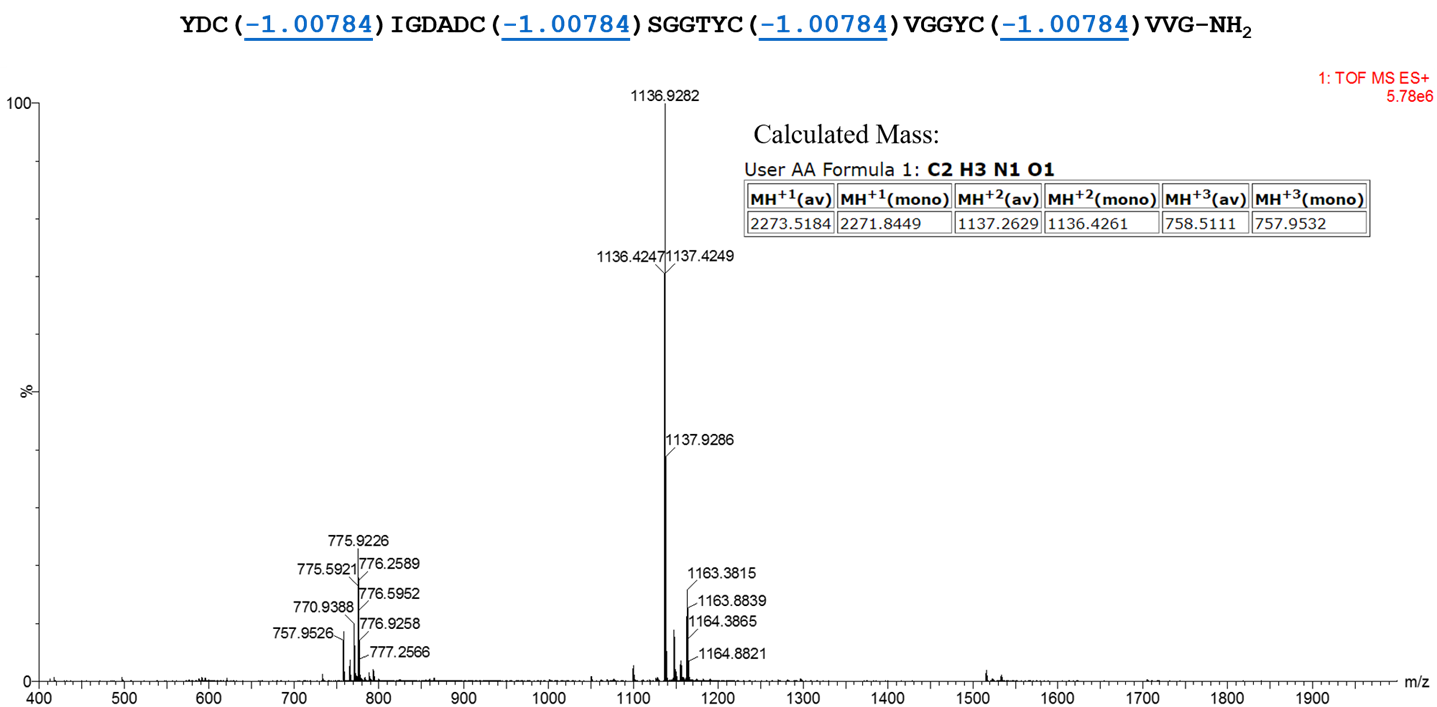


**Figure S2.** Mass spectrum of native cdg14a and calculated mass.


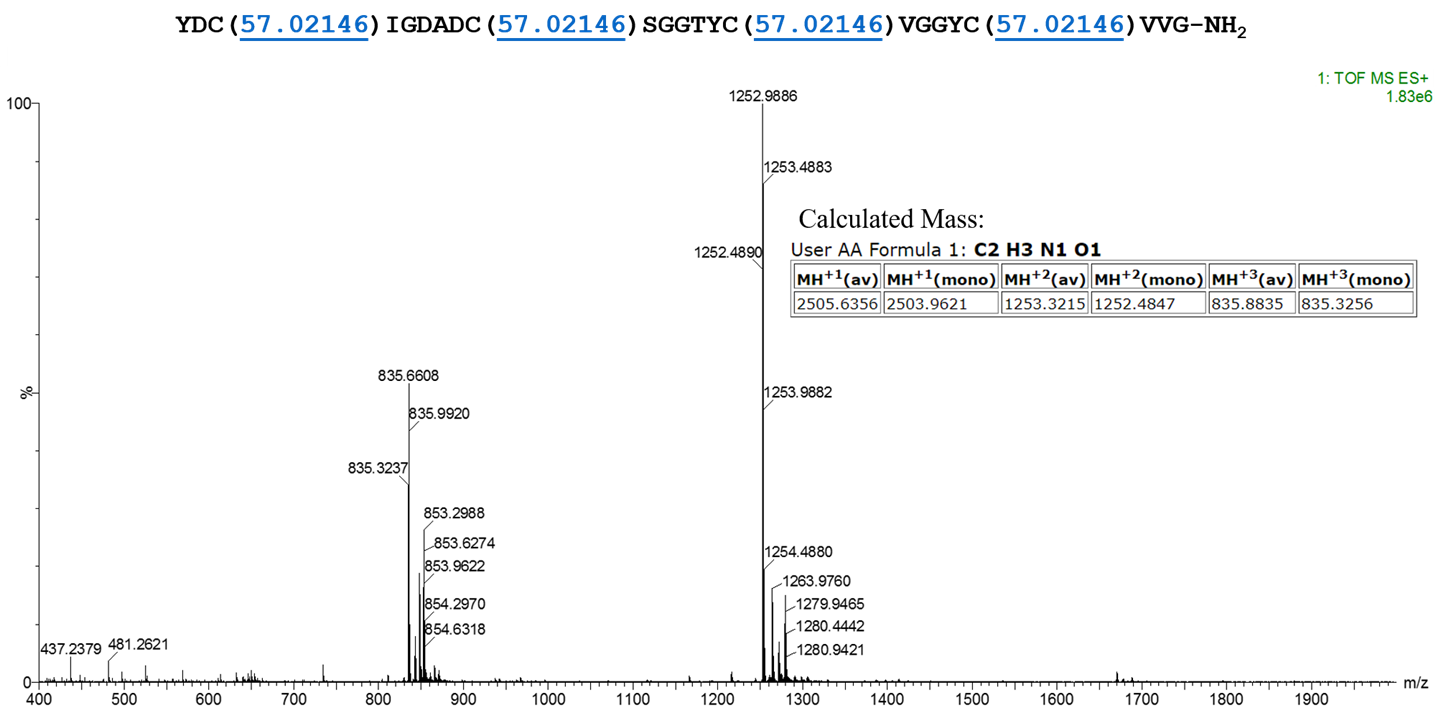


**Figure S3.** Mass spectrum of reduced (dithiothreitol) and alkylated (iodoacetamide) cdg14a and calculated mass.


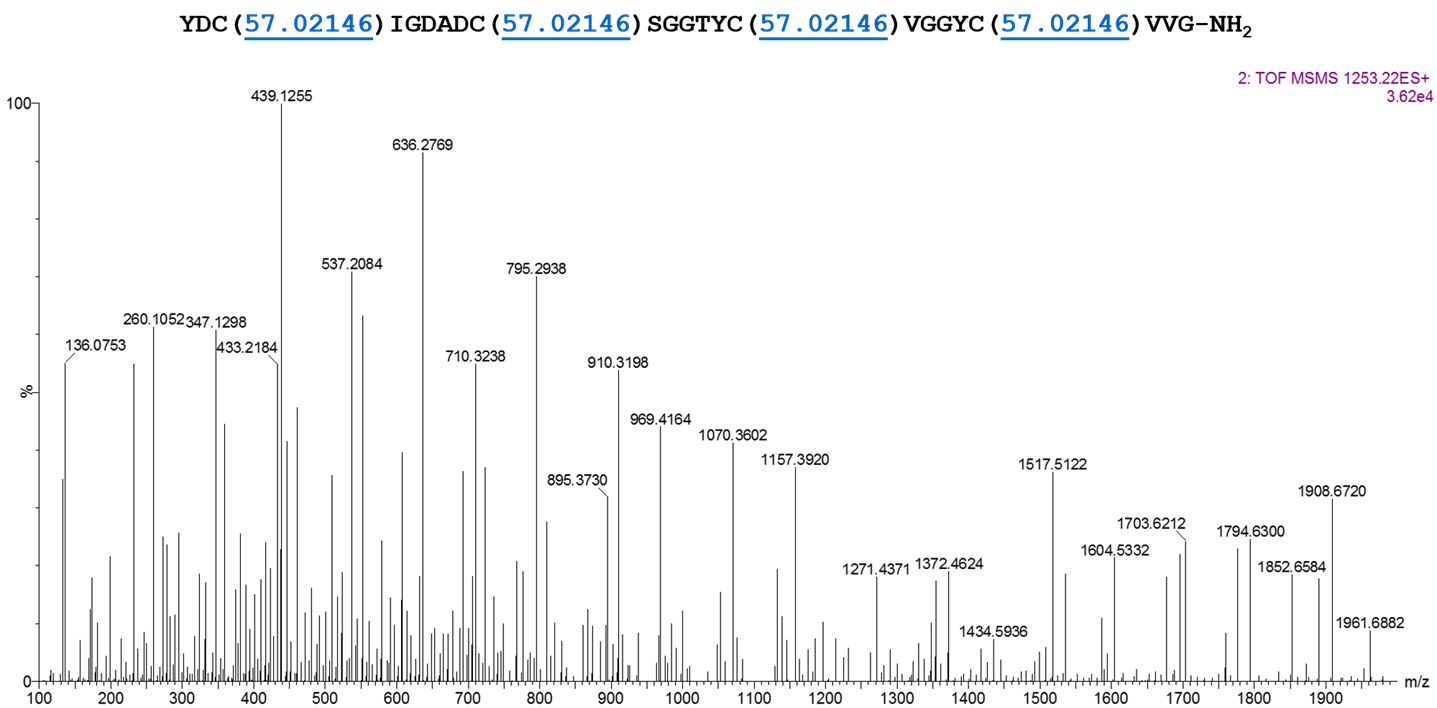


**Figure S4.** MS/MS fragmentation spectrum of reduced and alkylated cdg14a.


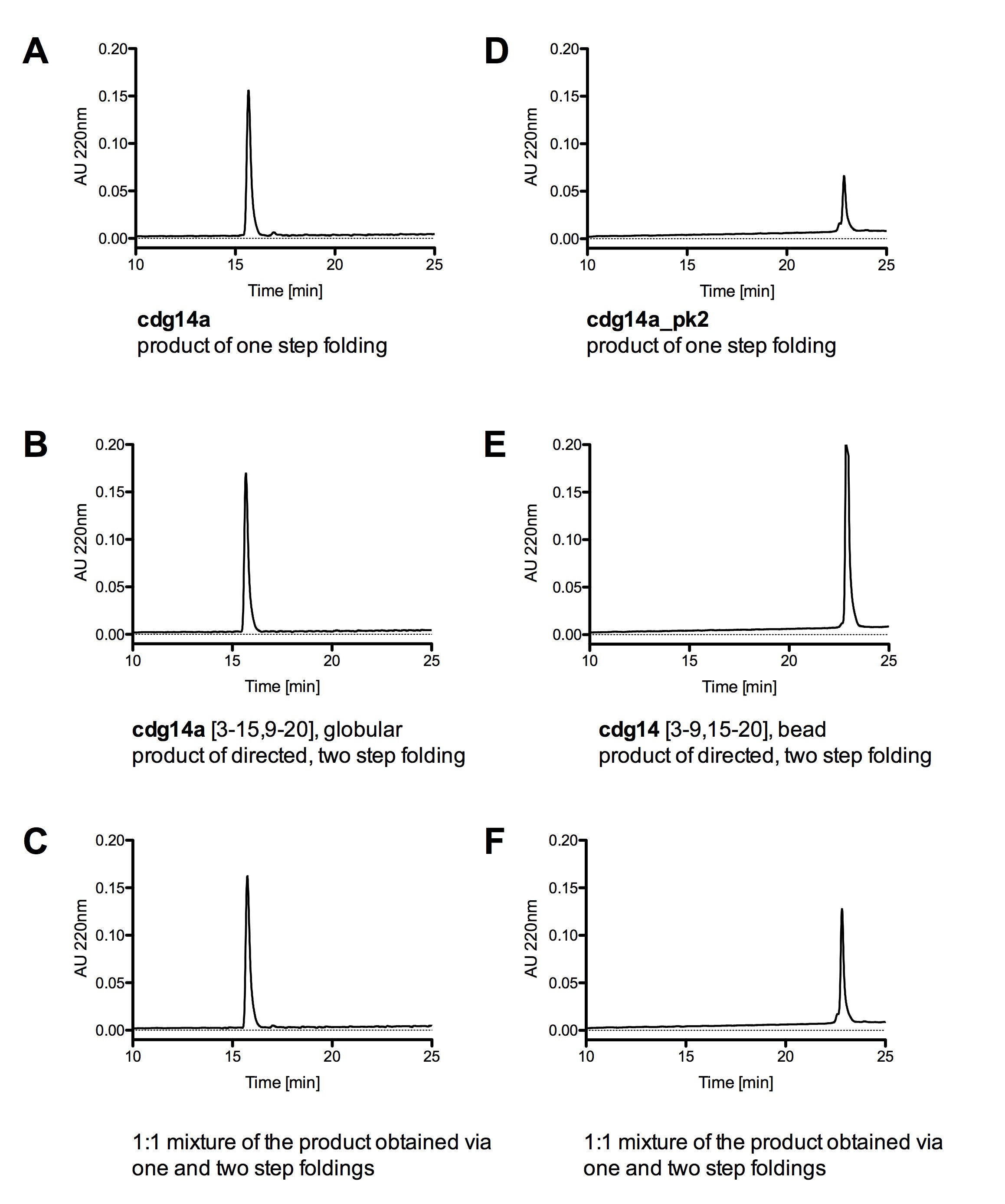


**Figure S5.** Co-elution experiment of the globular and bead forms prepared in one and two step folding. HPLC profiles of cdg14a obtained via one step, glutathione assisted folding (**A**), two-step directed folding (**B**), mixture of cd14a obtained using both methods (**C**) and cdg14a [3-9, 15-20] obtained via one step, glutathione assisted folding (**D**), two-step directed folding (**E**), mixture of cd14a obtained using both methods (**F**). Chromatograms were recorded using an analytical C18 column and a gradient ranging from 15% to 45% solvent B in 30 min with 1 ml/min flow rate. The HPLC solvents were 0.1% (vol/vol) TFA in water (solvent A) and 0.1% TFA (vol/vol) in 90% aqueous CH_3_CN (vol/vol) (solvent B). The eluent was monitored by measuring absorbance at 220 nm.
